# Burden of household food insecurity in urban slum settings

**DOI:** 10.1371/journal.pone.0214461

**Published:** 2019-04-02

**Authors:** Ashish Joshi, Arushi Arora, Chioma Amadi-Mgbenka, Nidhi Mittal, Shruti Sharma, Bhavya Malhotra, Ashoo Grover, Archa Misra, Menka Loomba

**Affiliations:** 1 Department of Epidemiology, City University of New York Graduate School of Public Health and Health Policy, New York, New York, United States of America; 2 Foundation of Health care Technologies Society, New Delhi, India; 3 Columbia University Mailman School of Public Health, New York, New York, New York, United States of America; 4 Indian Council of Medical Research, New Delhi, India; University of Texas Medical Branch at Galveston, UNITED STATES

## Abstract

This study examined the burden of food insecurity in India’s un-notified slums, using an SDG framework to identify correlates of food insecurity. A convenience sampling approach was employed in selecting 38 slums from 675 un-notified slums across four geographic zones. Ten percent of the households in each slum site were selected from each zone, and one household member was interviewed, based on their availability and fulfilment of the eligibility criteria. Eligible individuals included those aged 18 years and above, who were resident in the selected slums and provided consent. Individuals with mental or physical challenges were excluded. A total sample of 907 study participants were included. Results showed that 43% (n = 393) of the participants were food insecure. More than half were females (73%, n = 285), who had not completed any schooling (51%, n = 202). One-third (n = 128) resided in the Northern Region of Delhi. SDG-related predictors of food insecurity included: household educational level (*SDG 4 Quality education) (p = 0*.*03)*, coverage of health service needs (*SDG 3 Good health and well-being*) *(p = 0*.*0002)*, electricity needs (*SDG 7 affordable and clean energy*) (p<0.0001), and employment needs (*SDG 8 Decent and economic growth*) *(p = 0*.*003)*. Having healthcare needs that were partially or fully met was equally associated with higher food insecurity: this could be attributed to high healthcare costs and the lack of federal subsidies in un-notified slums, collectively contributing to high out-of-pocket health costs. Failure to fully meet employment needs was also significantly associated with higher food insecurity. However, met needs for electricity, finance, women’s safety and satisfactory family relationships, were associated with lower food insecurity. Household predictors of food insecurity included: number of household members, and the presence of physically disabled household members. Necessary interventions should include connecting food insecure households to existing social services such as India’s Public Distribution System, and multi-sector partnerships to address the existing challenges.

## Introduction

Fifty-five percent of the global population resides in urban areas and this estimate is projected to rise to 68% by 2050 [1]. This growth poses challenges in meeting the array of urban population needs related to housing, transportation, health, education, and employment [1]. Projections by the United Nations Population Division and the Department of Economic and Social Affairs indicate that urban population growth will be predominant in Africa and Asia [1]. In particular, India, China and Nigeria will experience the largest growth in urban populations and will account for thirty-five percent of global urban growth between 2018 and 2050 [1]. Managing urban areas, especially in populated countries has become one of the most critical development challenges of the 21st century [1]. Rapid urbanization has precipitated a proliferation of informal settlements and the development of new, smaller cities plagued by urban poverty [2]. The resulting smaller, marginalized cities, also known as “slums”, constitute the most prominent manifestations of urban poverty in developing countries.

The UN-Habitat defines slums as areas of people lacking one or more of the following indicators: durable housing of permanent nature, sufficient living space, easy access to safe water, access to adequate sanitation, and security of tenure [3]. According to the United Nations, slums are operationally defined as “groups of individuals living under the same roof in an urban area, lacking in one or more of the following five amenities: (a) Durable housing (b) Sufficient living area (c) Access to improved water (d) Access to improved sanitation facilities and (e) Secure tenure [3]. While the United Nations and UN habitat definitions of slum focus mostly on externally observable features such as housing structure, water, sanitation and security facilities; recent contextually relevant definitions of slums based on country-level studies from places such as India and Uganda, have included more specific characterization of slum settings and their residents. For instance, slums are portrayed as: areas with lack of basic services such as electricity, unhealthy living conditions, high density of low-income earners, unemployed persons with low literacy levels, child health challenges and undernutrition, high poverty and social exclusion, high noise levels, crime, drug abuse, immorality, alcoholism, high STD prevalence [4,5]. These descriptions are widely reflected in the Sustainable Development Goals (SDG) assessed in the present study including: SDG3 (good health and well-being), SDG4 (quality education), SDG6 (clean water and sanitation), SDG7 (affordable and clean energy), SDG8 (decent work and economic growth).

An estimated one billion people worldwide currently live in slums, making up a third of the world's urban population [1]. India’s urban population alone is projected to increase by over 400 million urban dwellers [1]. Consequently, India will face tremendous challenges in meeting the basic needs of its growing urban dwellers, including infrastructure, transportation, housing, energy, employment, education, healthcare, and food security [1]. Rapid proliferation in India’s urban slum population, alongside its marginalized workforce with predominantly casual or contract employment, constitute a food and nutrition emergency in urban India [6–10]. India performed poorly on the Global Hunger Index, a standardized tool reflecting severity in hunger levels by drawing on indicators of undernourishment, child wasting, child stunting and child mortality [11]. According to a 2018 Global Hunger Index report, India ranked at the 103^rd^ position (out of 119 countries), doing better than just 16 countries in terms of hunger severity [11]. Household food insecurity has resulted in a variety of ill effects including psychosocial dysfunction in children, socio-familial problems and overall poor health status [12]. Urban food insecurity requires critical attention since an important component of urbanisation is the proliferation of slums caused by the unplanned migration of the rural poor to urban areas in search of better livelihoods [10, 13].

Slum growth in India has been attributed to an increase in the informal workforce and a lack of basic health and hygiene facilities—two of the most frequently identified predictors of food insecurity in developing settings [6, 7]. These food insecurity correlates disproportionately affect un-notified slums in India. Notification constitutes the process of legally designating slum settlements as federally-recognized, with the goal of affording residents the rights to portable water and sanitation [14]. India’s government schemes aimed at improving welfare across slums have largely been focused on the upgrade of “notified” urban slums [7]. India’s un-notified slums which constitute about half of its total slum population, remain deprived of numerous benefits, notably subsidies by the Public Distribution System (PDS) of India, in the form of food and fuel [7–9]. Consequently, residents of these un-notified slums purchase their food at regular market prices since they lack these subsidies [8, 9]. Prior research on food insecurity has mostly focused on notified urban slums of India, with a dearth of research done in un-notified slums, owing to difficulty in accessing these populations [15].

The objective of this study was to examine the burden of food insecurity in India’s un-notified slums. This study utilized an SDG framework which involved visualizing potential food insecurity correlates in the context of the SDGs. Specifically, our approach in relation to assessing food insecurity determinants draws from the SDG Goal 2: “**Zero hunger**”, which embodies the provision of food security, nutrition, and sustainable agriculture [16]. In 2012, the Zero Hunger Challenge was launched at the United Nations Conference on Sustainable Development with the goal of fostering five key improvements including: “100 percent access to adequate food all year round; zero-stunted children under age 2; sustainability of food systems; 100 percent increase in smallholder productivity and income; and zero loss or waste of food”; all of which collectively capture the array of challenges related to food insecurity and malnutrition. [16].

Potential correlates of food insecurity (as identified in SDG goal 2 above) were assessed using related SDG indicators including: coverage of health services (SDG3 good health and well–being), educational and ICT skills (SDG4 quality education), owning a cell phone (SDG5 gender equality), availability of toilet facility (SDG6 clean water and sanitation), electricity needs (SDG7 affordable and clean energy) and employment needs (SDG8 decent work and economic growth), among vulnerable populations living in India’s un-notified slum settings. In addition, we assessed several markers of social disadvantage based on their established relationship with food insecurity across marginalized populations, especially among women [5, 17–19]. These variables included: financial needs, satisfactory family relationships, general safety needs, women safety and child health and education needs. India’s un-notified slum settings which have been rarely studied provides an ideal environment to examine food insecurity correlates using an SDG framework.

### SMAART informatics framework

**Fig 1:**
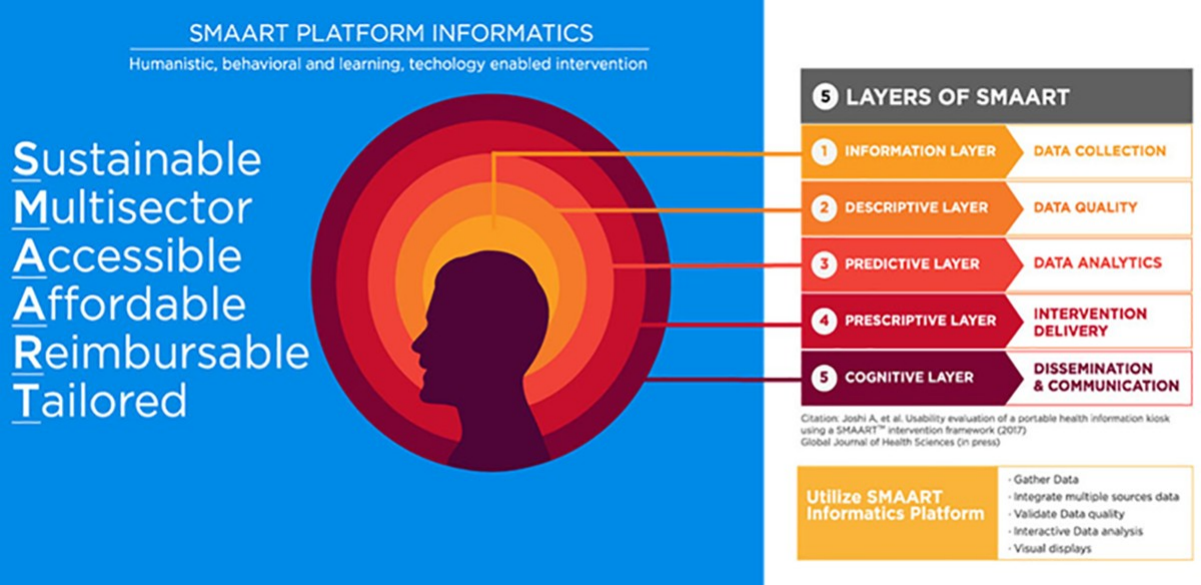
SMAART Informatics Components. This figure represents the different layers of the SMAART platform.

We utilized an informatics framework known as SMAART (S-Sustainable, M-Multisector, A-Accessible, A-Affordable, R-Reimbursable, T-Tailored) to assess the SDG-related food insecurity correlates across urban slum settings in India. SMAART is a Population Health Informatics (PopHI) framework that was designed using the principles of Data, Information and Knowledge (DIK), Human Centered approach, Information processing theory and humanistic, behavioral and learning theories. The framework integrates social determinants of health to facilitate informed decision making (Fig 1). Details of the SMAART framework have been previously published [13].

**Fig 2:**
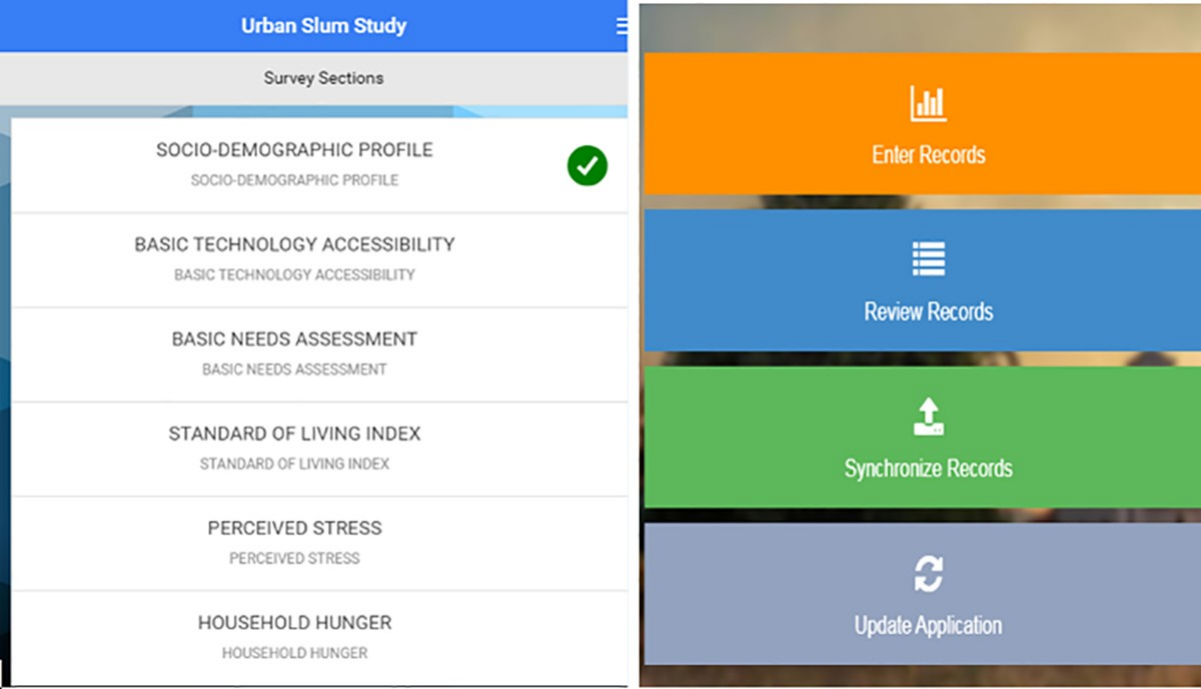
SMAART platform overview. This figure represents the landing page of the application and the data entry sections available.

**Fig 3:**
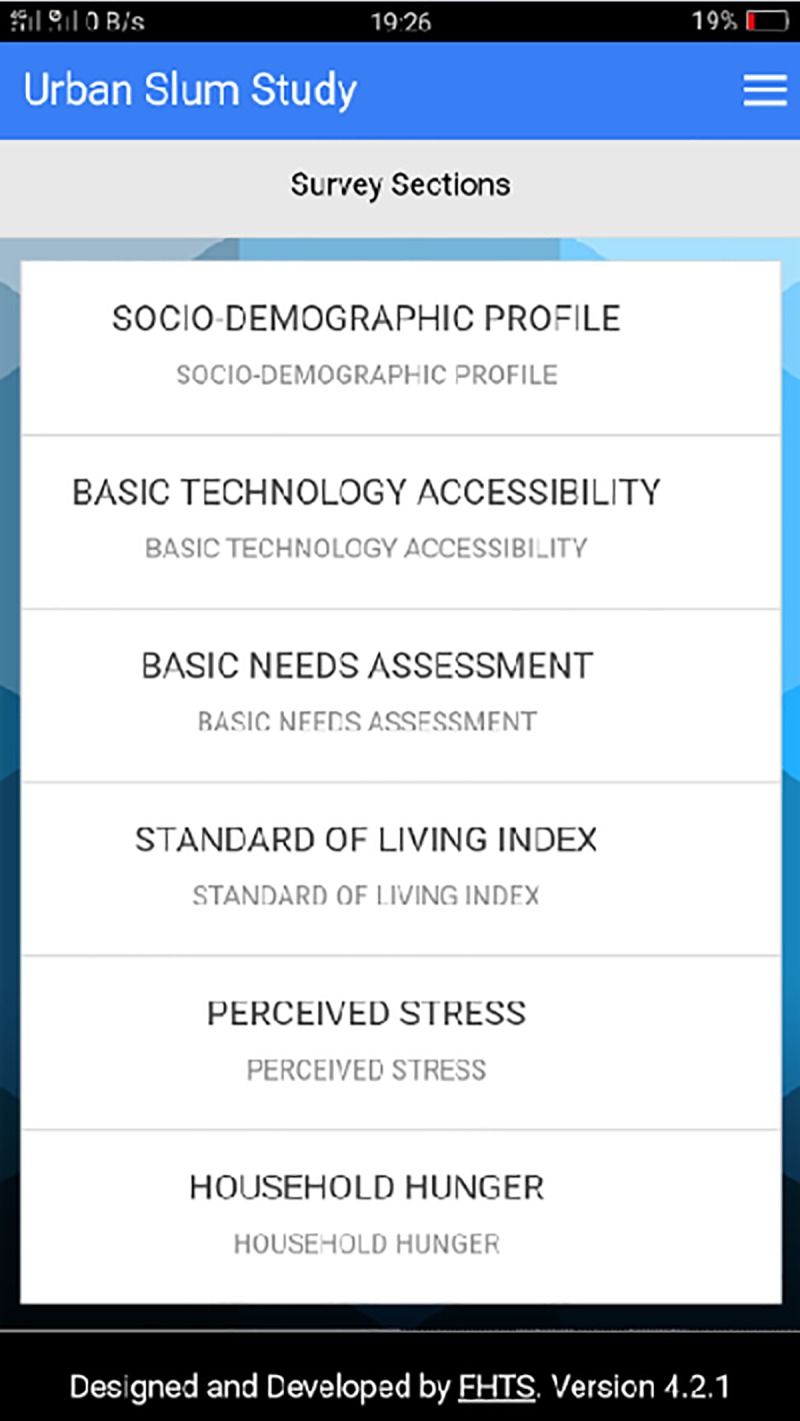
Survey sections of the SMAART platform. This figure represents the different survey sections comprise the variable categories assessed.

**Fig 4:**
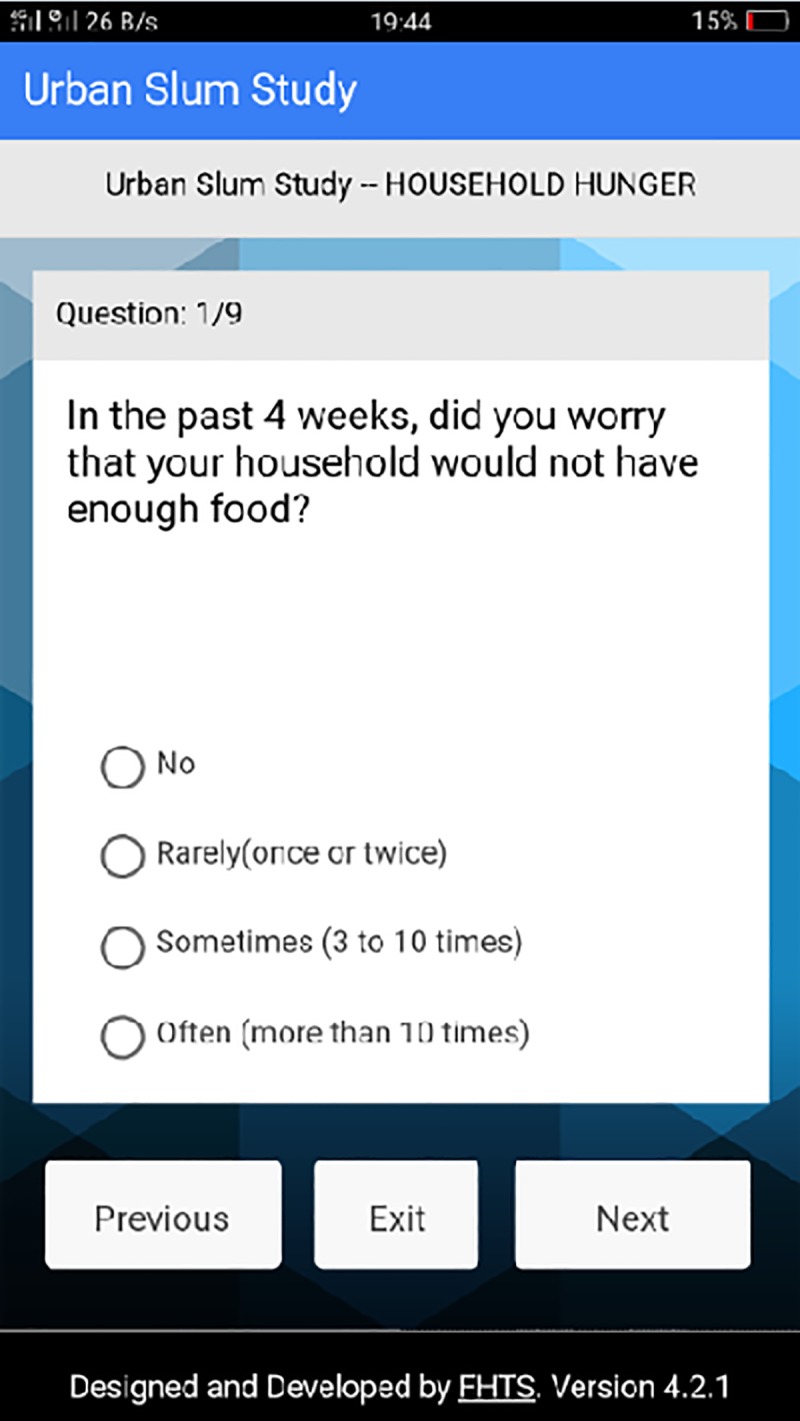
Question 1 on household hunger section. This page of the platform represents the first question asked in the data section for household hunger.

**Fig 5:**
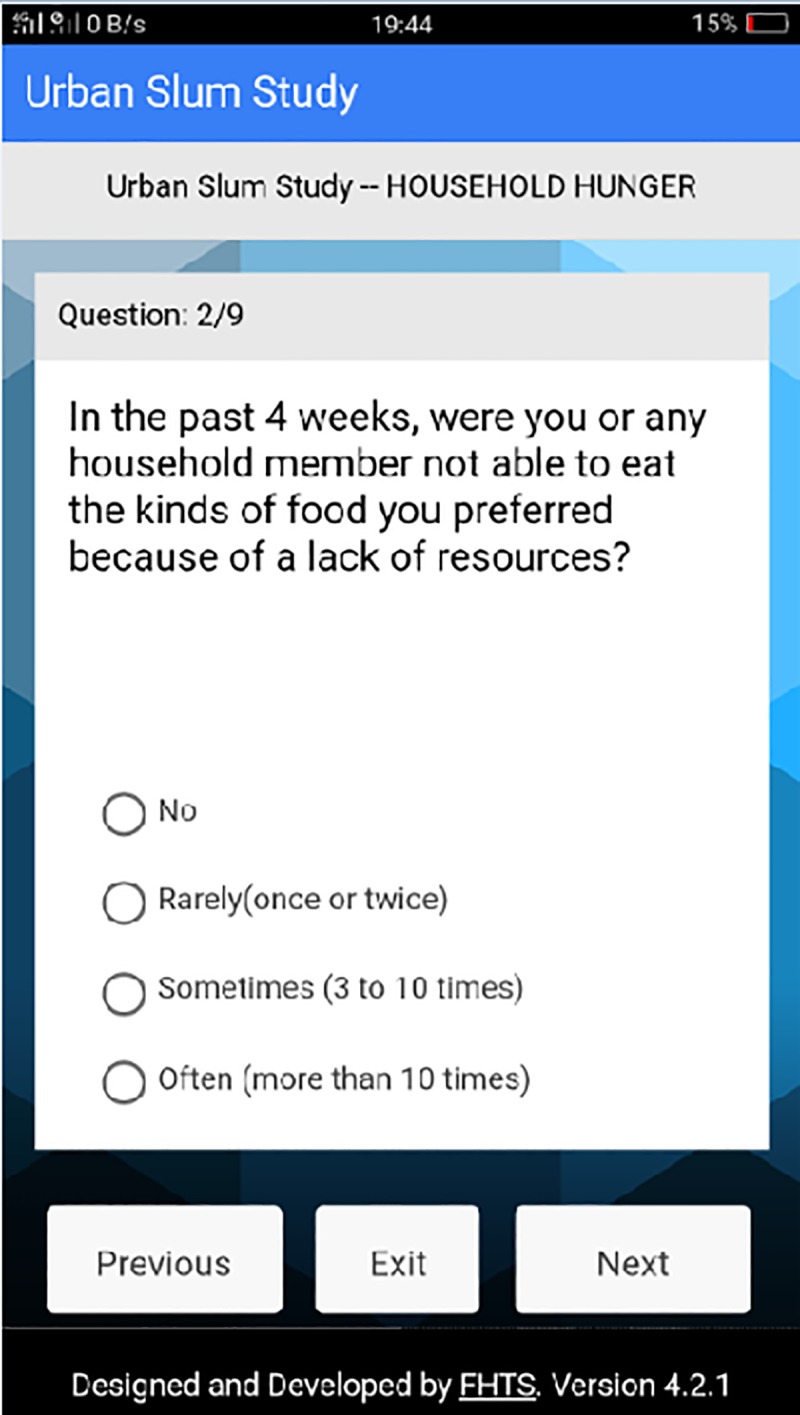
Question 2 on household hunger section. This page of the platform represents the second question asked in the data section for household hunger.

**Fig 6:**
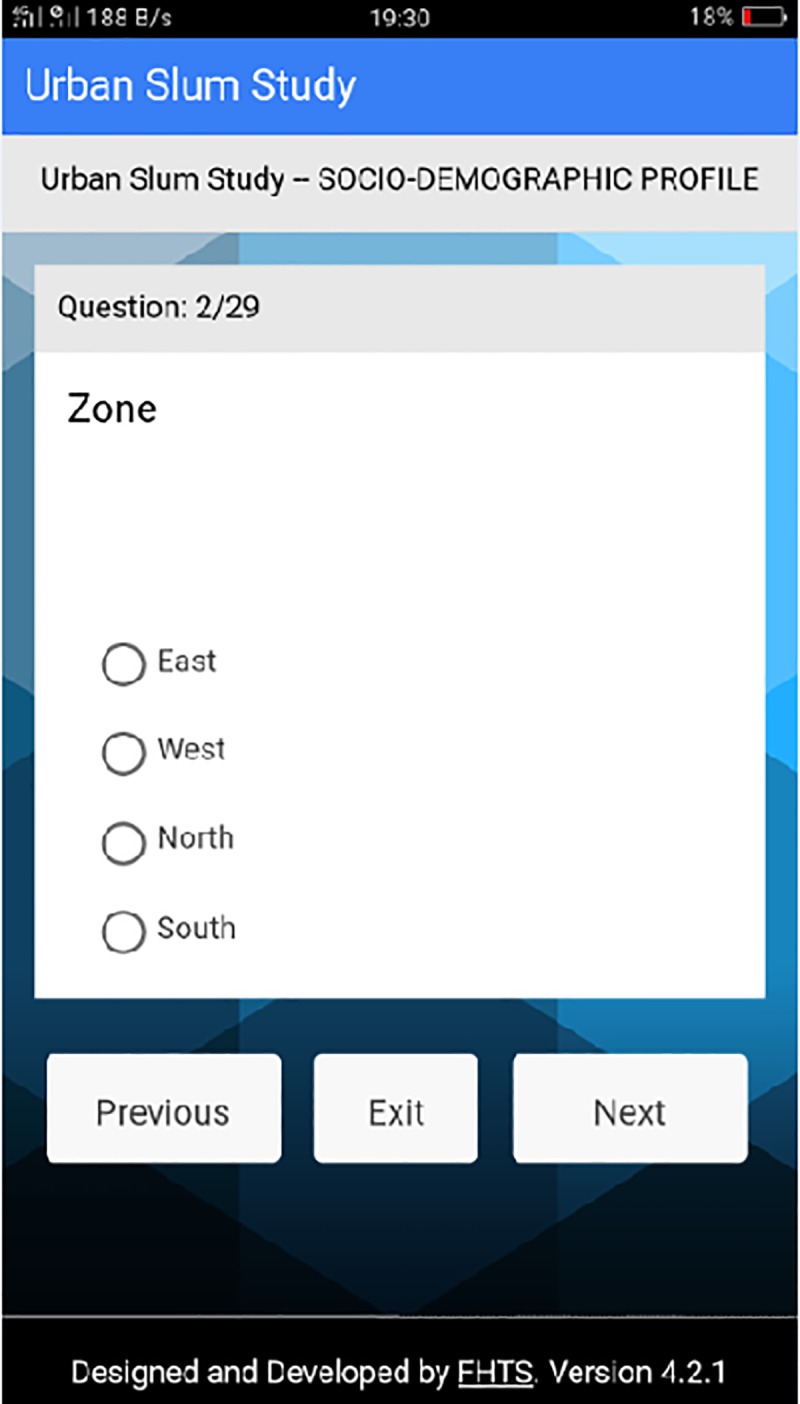
Question 2 on socio-demographic profile. This page of the platform represents the second question asked in the data section for socio-demographics.

**Fig 7:**
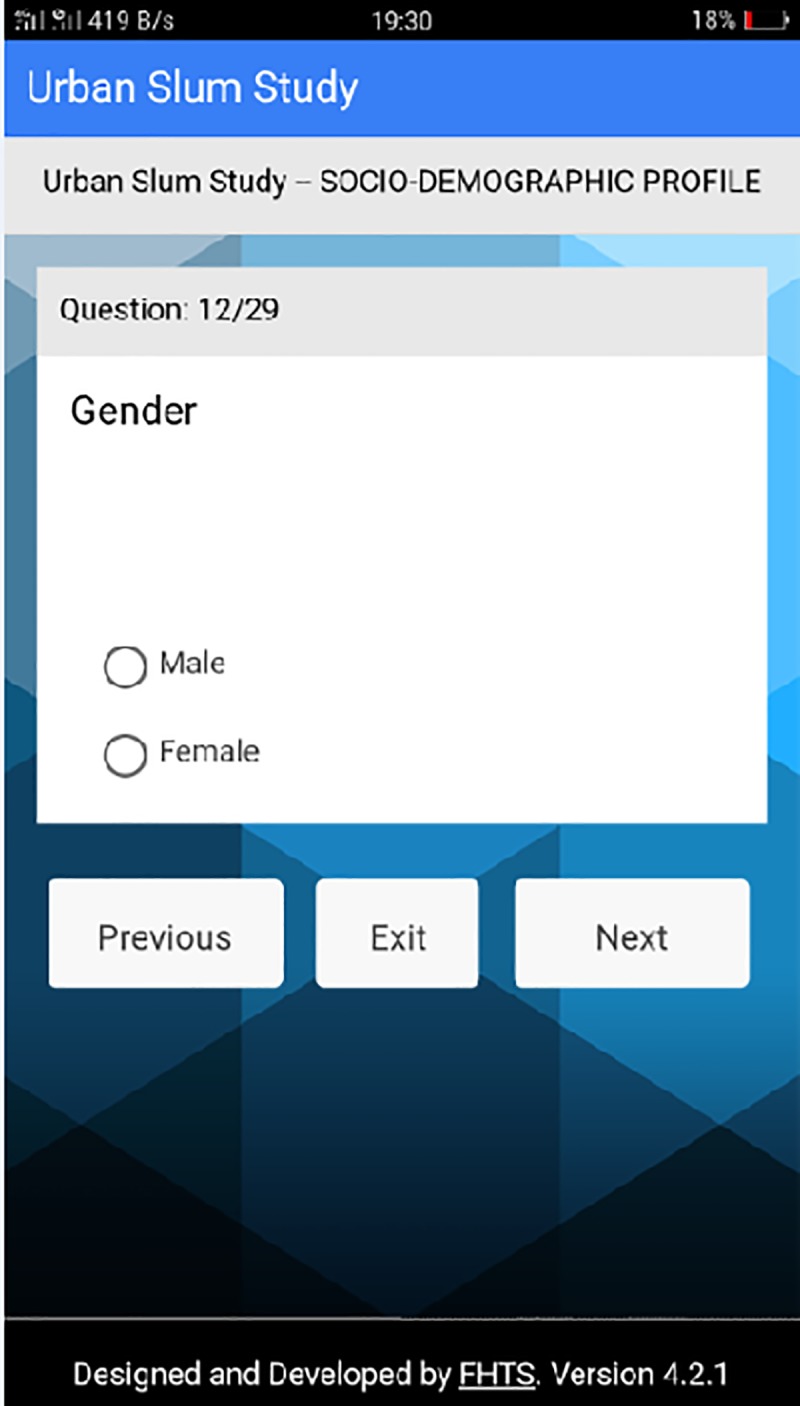
Question 12 on socio-demographic profile. This page of the platform represents the twelfth question asked in the data section for socio-demographics.

The SMAART framework facilitates: (a) transmission of data regarding the health status of the consumer, (b) interpretation of data in an evidence-based manner (c) addressing specific consumer needs, (d) provision of timely feedback to the consumer and (e) repetition of the feedback loop of information processing (Fig 1). The SMAART informatics platform collects, processes and presents social determinants of population health data, in a meaningful and contextually relevant format that is easy to understand. This framework can be operationalized as either an interactive standalone system, or internet-enabled platform. In the current study, we operationalized SMAART in the form of an android mobile data collection platform which was utilized in gathering data on several variables including socio-demographics, technology access and familiarity, SDG indicators, and household food insecurity (Figs 2–7). The collected data was stored in a password protected Microsoft SQL database and was then uploaded via the internet to a Linux server.

## Materials and methods

**Fig 8:**
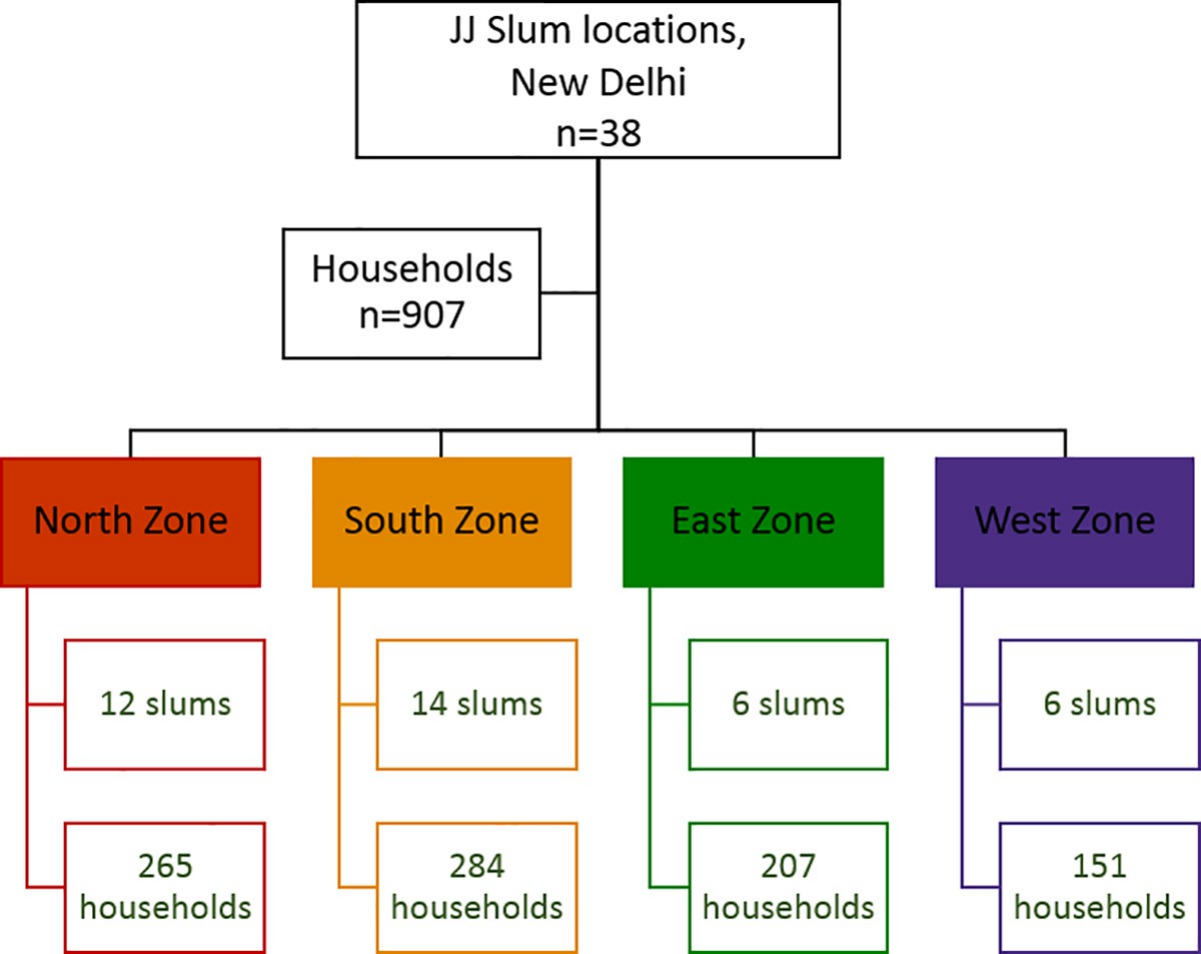
Study participant recruitment. This figure represents the study participant recruitment process.

A cross-sectional study was conducted between June 2016 and January 2017 to assess the burden of food insecurity in urban slums of Delhi, India. A convenience sampling approach was utilized, and this involved sample selection based on the judgement of the researcher [4]. The sampling frame utilized was the “Delhi Urban Shelter Improvement Board Jhuggi-Jhopadi Cluster List of 2015”, which enumerated a total of 675 un-notified urban slums across the four geographic zones (North, South, East, West) of New Delhi, India. From the 675 un-notified slums, we selected 38 slum sites across 4 zones (North zone = 12, South zone = 14, East zone = 6, West zone = 6) (Fig 8). From each zone, we then selected 10 percent of the households based on proximity to the researcher, ease of access to the slums, and the presence of local collaborators who could help in navigating the slums. One member from each household was selected and interviewed, based on their availability and fulfilment of the eligibility criteria. Eligible individuals included those who were aged 18 years and above, resident in these slums and provided voluntary consent to participate in the study. Individuals who had mental or physical challenges were excluded from the study. This resulted in a total sample of 907 study participants across all the slums. All the participants provided written informed consent (response rate of 100%). The study protocol was approved by the Institutional Review Board of the Foundation of Healthcare Technologies Society, New Delhi, India (IRB number: #FHTS/041/2016).

The Household Food Insecurity Access Scale was used as a proxy measure for SDG 2-Zero Hunger. Household Food Insecurity was assessed using the Household Food Insecurity Access Scale (HFIAS) (S1 Appendix) [20]. This scale consists of nine (9) questions with a focus on participants experiences with food scarcity, the associated inconvenience, and their behavioural responses to food insecurity [20]. Each of these questions inquire whether a particular condition of food insecurity is experienced in a household (yes/no), as well as its frequency of occurrence (rarely/sometimes/often) [20]. Response scores range from a minimum of 0 to a maximum of 27. These questions serve to assign households along a continuum of severity from food secure to food insecure, and to monitor changes in household food insecurity over time [20]. The questions are based on a recall period of four weeks. The HFIAS categorizes households as being increasingly food insecure based on their affirmative responses to more severe food insecurity conditions (S1 Appendix). Households in this study were classified into four groups according to the HFIAS (S1 Appendix). They include: Food Secure, Mildly Food Insecure, Moderately Food Insecure and Severely Food Insecure, as described below [20]:

- Food Secure: Any household which has not experienced any of the food insecurity conditions listed, or just experiences worry, but rarely.
- Mildly Food Insecure: Any household that worries about not having enough food—sometimes or often, and/or is unable to eat preferred foods, and/or eats a more monotonous diet than desired, and/or some foods considered undesirable, but only rarely. Households classified as mildly food insecure do not cut back on food quantity and do not experience the three most severe conditions of food insecurity: running out of food, going to bed hungry, or going a whole day and night without eating.
- Moderately Food Insecure: Any household that sacrifices quality more frequently, by eating a monotonous diet or undesirable foods—sometimes or often, and/or has started to cut back on quantity by reducing the size of meals or number of meals—rarely or sometimes. Households classified as moderately food insecure do not experience any of the three most severe conditions of food insecurity described above.
- Severely Food Insecure: Any household that cuts back on meal size or number of meals often, and/or experiences any of the three most severe food insecurity conditions (running out of food, going to bed hungry, or going a whole day and night without eating), even rarely [20].

Dichotomous categories comprising of food secure and insecure households were created from the above groups. Specially, the three categories: mildly food insecure, moderately food insecure and severely food insecure were combined into a new group (food insecure), and this was compared with the food secure group.

### Variables gathered

- Socio-demographics: Information was collected on participants age, gender, educational status, occupation, household size, family size, and number of earning members in the household.
- Technology access and familiarity: Information was collected about cell-phone ownership in households, type of cell phone, access to internet, and knowledge of text messaging.
- Sustainable Development Goals (SDGs): Information was collected about the participants perceptions regarding coverage of several SDG-related food insecurity correlates including healthcare service needs, educational needs, employment needs, financial needs, family relationships, water, sanitation and hygiene needs, drinking water needs, electricity needs, general safety, women safety needs, and child health and education needs. The Perceived Need for Care Questionnaire (PCNQ), which examines individual perceptions of various health and social service needs was adapted for use in the present study to assess the study participants’ levels of perceived need for the SDG-related food insecurity correlates. The levels of perceived need included: met need, partially met/somewhat met need, and unmet need. A met need implies that a perceived service was received to the extent that it was needed; a partially or somewhat met need refers to a service that was received but the intervention was insufficient in meeting expectations; while an unmet need is a service that was needed but not received [21].
  ○ SDG 2 (Zero Hunger): The Household Food Insecurity Access Scale was used as a proxy measure for SDG 2-Zero Hunger. Details on the HFIAS have been provided in the methods section (S1 Appendix).
  ○ SDG 3 (Good Health and Well-Being): This was evaluated based on the extent to which healthcare services were perceived to be met.
  ○ SDG 4 (Quality education): This was evaluated based on current educational status and the extent to which educational service needs were perceived to be met.
  ○ SDG 5 (Gender Equality): Gender differences in food insecurity levels were also assessed.
  ○ SDG 6 (Clean Water and Sanitation): This was evaluated based on the extent to which water, sanitation and hygiene, and drinking water needs were perceived to be met.
  ○ SDG 7 (Affordable and Clean Energy): This was evaluated based on the extent to which electricity needs were perceived to be met.
  ○ SDG 8 (Decent work and economic growth): This was evaluated based on extent to which employment needs were perceived to be met.
  ○ Additional variables that were assessed—based on the levels of perceived need included financial needs, satisfactory family relationships, general safety needs, women safety and child health and education needs. The influence of mentally ill, physically disabled, or permanently ill household members on food insecurity was also assessed.

### Statistical analysis

Descriptive analysis was conducted to report means and proportions for the continuous and categorical variables respectively. The outcome, food insecurity, was examined as both a continuous and categorical variable. Bivariate analyses using T-tests and ANOVA were conducted to compare the average food insecurity scores across the independent continuous and categorical variables. A stratified analysis was then conducted to examine the distribution of food insecurity (as a binary variable-food secure vs insecure) across the various demographic and SDG-related indicators. Chi-square tests of significance was used to examine the differences across groups. A Multivariable logistic regression model was then fit to identify predictors of food insecurity, using variables that were significant in the bivariate models. Results were reported to be significant at a p-value<0.05. Analysis was performed using SAS V 9.4.

## Results

### Study sample characteristics

The average age of the study participants was 36 years (SD = 13). The majority of them were females (66%, n = 602). Forty-six percent of the study participants had incomplete schooling (n = 420), and 71% (n = 643) of them had 5 or more household members (Table 1). The highest level of education attained in the household was less than a high school diploma, among 50% (n = 455) of the respondents. There were two earning members on average per household. Forty-nine percent of study participants worked for 7–8 hours per day (Table 1). Sixty percent (n = 599) of the study participants reported owning a phone while 34% (n = 304) did not own a phone (landline or mobile) (Table 1).

**Table 1:** Study sample characteristics.

Socio-demographics	Results
Age, years	Mean = 36; SD = 13
Gender, Females	66% (N = 602)
Total number of Household Members	
1	2% (N = 16)
2 to 4	27% (N = 249)
5 or more	71% (N = 643)
Education level of Respondent	
No school	41% (N = 376)
Incomplete school	46% (N = 420)
High school	7% (N = 62)
Some college	1% (N = 12)
Graduate/ Post-graduate	4% (N = 37)
Highest level of education attained by household	
No School	16% (N = 142)
Incomplete school	50% (N = 455)
High school diploma	17% (N = 155)
Some college	5% (N = 49)
Graduate/ Post-graduate	12% (N = 106)
Earning members in household	Mean = 2; SD = 1
Number of working hours/day of the earning member	
None	6% (N = 48)
6 hours or less	13% (N = 113)
7 to 8 hours	49% (N = 422)
9 to 10 hours	15% (N = 132)
More than 10 hours	16% (N = 141)
Zone	
West	17% (N = 151)
East	23% (N = 207)
North	29% (N = 265)
South	31% (N = 284)
** Access to Technology**	
Own a Phone	
No	34% (N = 304)
Yes	66% (N = 603)
Knowledge of texting	
No	51% (N = 459)
Yes	49% (N = 448)
Internet Access	
No	76% (N = 686)
Yes	24% (N = 221)

### Indicators of sustainable development goals

More than half of the study participants reported that their educational needs (52%, n = 474), family relationships (55%, n = 495), women safety (54%, n = 485), child health and educational needs (53%, n = 477) had been met. Half of the study participants reported that their general safety needs had been met (n = 457). More than half of the study participants perceived that their financial needs were somewhat met (56%, n = 512). About one-third of the study participants perceived that their employment needs (31%, n = 281), water and sanitation hygiene (37%, n = 330), drinking water (28%, n = 257) and healthcare services (25%, n = 227) needs were not met at all (Table 2).

**Table 2:** Levels of perceived need among study participants, based on the SDG indicators assessed.

Indicators of SDGs	% (N)
**SDG3 Good health and well-being**	
***Healthcare Service needs***	
Met	39% (N = 350)
Somewhat met	36% (N = 330)
Not met at all	25% (N = 227)
**SDG4 Quality education**	
Educational needs*	
Met	52% (N = 474)
Somewhat met	30% (N = 268)
Not met at all	18% (N = 163)
**SDG8 Decent work and economic growth**	
Employment needs*	
Met	32% (N = 293)
Somewhat met	37% (N = 332)
Not met at all	31% (N = 281)
**SDG6 Clean water and sanitation**	
*Water*, Sanitation and Hygiene needs*	
Met	26% (N = 237)
Somewhat met	37% (N = 338)
Not met at all	37% (N = 330)
Drinking Water needs*	
Met	37% (N = 331)
Somewhat met	35% (N = 318)
Not met at all	28% (N = 257)
**SDG7 Affordable and Clean Energy**	
Electricity needs*	
Met	78% (N = 707)
Somewhat met	12% (N = 113)
Not met at all	10% (N = 86)
***Financial needs***	
Met	26% (N = 233)
Somewhat met	56% (N = 512)
Not met at all	18% (N = 162)
***Satisfactory family relationships***	
Met	55% (N = 495)
Somewhat met	33% (N = 303)
Not met at all	12% (N = 109)
***General Safety needs***	
Met	50% (N = 457)
Somewhat met	28% (N = 254)
Not met at all	22% (N = 196)
Woman Safety needs*	
Met	53% (N = 485)
Somewhat met	27% (N = 248)
Not met at all	20% (N = 173)
Child's Health and education needs*	
Met	53% (N = 477)
Somewhat met	33% (N = 304)
Not met at all	14% (N = 125)

“*” indicates variables with missing values

### Household food insecurity

**Fig 9:**
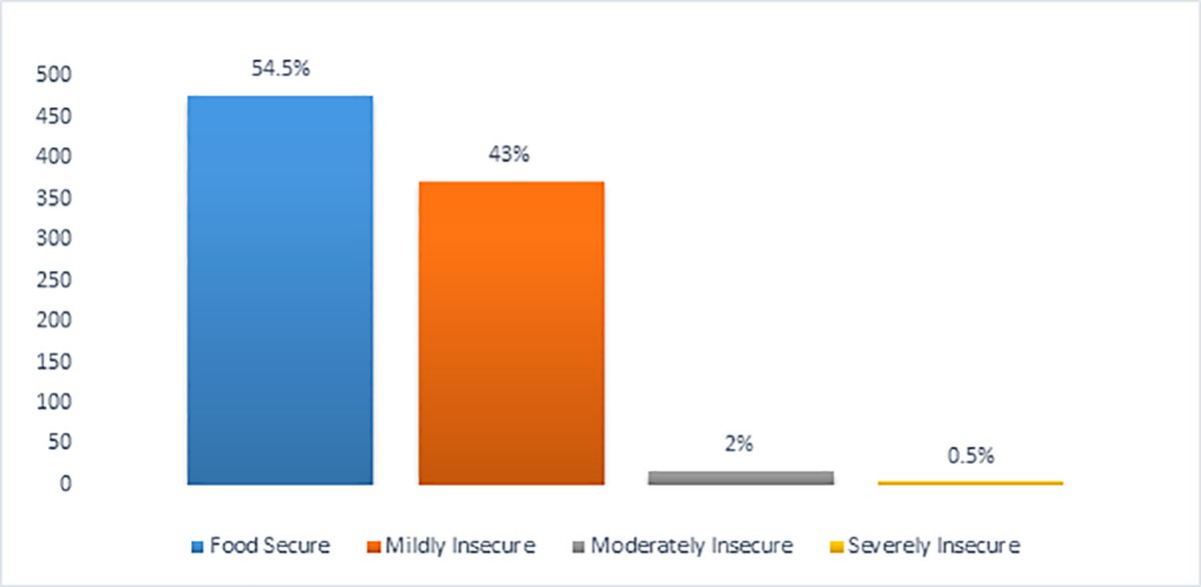
Percentage distribution of respondents into various categories of food security. This figure describes the proportion of participants in each food insecurity category.

More than half of the study participants were food secure (55%, n = 476). Less than 1% of the study participants were severely food insecure (0.5%, n = 4) (Fig 9).

### Association between socio-demographics and household food insecurity

Household food insecurity scores were higher among females (Mean = 6.0, SD = 8.1), those with no schooling (Mean = 7.7, SD = 9.0), those having no earning members in the household (Mean = 15.1, SD = 9.7), and those working for more than 10 hours a day (Mean = 11.8; SD = 9.6). Food insecurity scores were also higher among study participants residing in the Northern Zone of New Delhi (Mean = 7.5, SD = 9.0) (Table 3). Age of study participants was not significantly associated with food insecurity (p = 0.72) (Table 3).

**Table 3:** Average household food insecurity scores by demographics.

Socio-demographics	Mean Household Food Insecurity scores (SD)	p-value
*Age*, *years*	7.5 ± 9.6	*0*.*72*
*Gender*		*0*.*01*
Female	6.0 ± 8.1	
Male*	4.5 ± 7.7	
	
*Total number of household members*	3.6 ± 6.3	*<* .*0001*
*Education level of respondent*		< .0001
No School*	7.7 ± 9.0	
Incomplete school	3.8 ± 6.8	<0.0001
High school diploma	4.3 ± 6.6	0.0015
Some college	5.0 ± 8.0	0.2381
Graduate/ Post-graduate	3.5 ± 7.2	0.0025
*Highest level of education attained by household*		*<* .*0001*
No school*	9.8 ± 9.5	
Incomplete school	4.8 ± 7.6	<0.0001
High school	5.2 ± 7.5	<0.0001
Some college	5.2 ± 6.9	0.0007
Graduate/ Post-graduate	2.8 ± 6.3	<0.0001
*Earning members in household*	4.3 ± 7.5	0.0007
*Number of working hours of the earning member*		*<* .*0001*
None*	15.1 ± 9.7	
6 hours or less	5.6 ± 7.3	<0.0001
7 to 8 hours	2.5 ± 4.9	<0.0001
9 to 10 hours	5.1 ± 7.8	<0.0001
More than 10 hours	11.8 ± 9.6	0.0055
*Zone*		*<* .*0001*
West*	3.2 ± 5.4	
East	6.8 ± 9.3	0.01
North	7.5 ± 9.0	0.0005
South	3.2 ± 5.4	0.06

“*” indicates the reference group.

### Stratified analysis

Stratified analysis was performed to compare study participants who were food secure to those who were food insecure. Study participants reporting mild, moderate and severe food insecurity were categorized as being food insecure. Results showed that nearly 40% (n = 393) of the study participants had some form of food insecurity. More than half of them were females (73%, n = 285), and had no schooling (51%, n = 202). About half of them were from households where most individuals had not completed high school (48%, n = 190). Thirty-four percent of them worked for 7 to 8 hours per day (n = 128). Thirty-three percent of these households were located in the Northern Region of New Delhi, India (n = 128) (Table 4).

**Table 4:** Percentage distribution of household food insecurity scores by demographics.

Socio-demographics	Total, N = 869 (%)		p-value
	Food Security* (N = 476)	Food Insecurity* (N = 393)	
**Age, years**	Mean = 38; SD = 12.7	Mean = 36; SD = 13.1	0.9946
**Gender**			0.0001
Female	60% (N = 287)	73% (N = 285)	
Male	40% (N = 189)	27% (N = 108)	
**Number of Household Members**	Mean = 6; SD = 2.7	Mean = 7; SD = 3.8	< .0001
**Education level of Respondent**			< .0001
No School	34% (N = 161)	51% (N = 202)	
Incomplete school	52% (N = 247)	39% (N = 152)	
High school diploma	7% (N = 34)	7% (N = 26)	
Some college	2% (N = 8)	1% (N = 4)	
Graduate/ Post-graduate	5% (N = 26)	2% (N = 9)	
**Education level of household**			< .0001
No school	11% (N = 53)	23% (N = 89)	
Incomplete school	52% (N = 246)	48% (N = 190)	
High school	16% (N = 79)	17% (N = 67)	
Some college	5% (N = 22)	6% (N = 22)	
Graduate/ Post-graduate	16% (N = 76)	6% (N = 25)	
**Earning members in household**	Mean = 2; SD = 1.1	Mean = 1; SD = 1	0.0016
**Number of working hours**			< .0001
None	2% (N = 8)	10% (N = 39)	
6 hours or less	12% (N = 53)	14% (N = 55)	
7 to 8 hours	63% (N = 284)	34% (N = 128)	
9 to 10 hours	14% (N = 67)	15% (N = 57)	
More than 10 hours	9% (N = 41)	26% (N = 100)	
**Zone**			0.0074
West	17% (N = 80)	18% (N = 71)	
East	24% (N = 115)	23% (N = 92)	
North	24% (N = 113)	33% (N = 128)	
South	35% (N = 168)	26% (N = 102)	

“*” indicates variables with missing values (n = 38). Analysis was conducted using complete observations for the food insecurity variable (HFIAS).

### Association between SDG indicators and household food insecurity scores (bivariate)

Household food insecurity was significantly associated with all the SDG indicators for basic needs, and other related variables assessed (p<0.05) (Table 5). Healthcare services, educational, employment, financial, satisfactory family relationships, water, sanitation and hygiene, drinking water, electricity, general safety, child health and education needs were significantly associated with food insecurity (p<0.05). (Table 6).

**Table 5:** Average household food insecurity scores by basic needs.

Indicators of SDGs	Mean Household Food Insecurity Score (SD)	p-value
Healthcare Services Needs		< .0001
Met	3.9 ± 6.5	<0.0001
Somewhat met	5.6 ± 7.8	0.0015
Not met at all*	7.8 ± 9.6	
Educational needs		< .0001
Met	3.5 ± 6.2	<0.0001
Somewhat met	6.0 ± 8.0	< .0001
Not met at all*	10.4 ± 9.9	
Employment Needs		< .0001
Met	2.3 ± 5.1	<0.0001
Somewhat met	7.1 ± 7.9	0.8224
Not met at all*	7.0 ± 9.5	
Financial Needs		< .0001
Met	1.1 ± 3.9	<0.0001
Somewhat met	5.2 ± 7.0	<0.0001
Not met at all*	12.9 ± 9.9	
Satisfactory family relationships		< .0001
Met	2.6 ± 5.3	< .0001
Somewhat met	6.1 ± 7.7	<0.0001
Not met at all*	17.1 ± 8.3	
Water, Sanitation and Hygiene needs		< .0001
Met	2.9 ± 5.7	0.0029
Somewhat met	5.5 ± 7.4	<0.0001
Not met at all*	7.4 ± 9.4	
Drinking Water needs		< .0001
Met	2.9 ± 5.4	<0.0001
Somewhat met	5.6 ± 7.7	<0.0001
Not met at all*	8.6 ± 9.8	
Electricity Needs		< .0001
Met	3.3± 5.8	<0.0001
Somewhat met	9.9 ± 9.3	<0.0001
Not met at all*	18.1 ± 7.9	
General Safety Needs		< .0001
Met	3.1 ± 5.6	<0.0001
Somewhat met	6.0 ± 8.1	<0.0001
Not met at all*	10.4 ± 10.0	
Woman Safety Needs		< .0001
Met	3.3 ± 5.9	<0.0001
Somewhat met	4.5 ± 7.4	<0.0001
Not met at all*	12.8 ± 9.6	
Child's Health and education need		< .0001
Met	3.1 ± 5.5	<0.0001
Somewhat met	5.6 ± 7.7	< .0001
Not met at all*	14.5 ± 9.7	
Mentally ill		0.0056
No*	5.3 ± 7.9	
Yes	9.2 ± 10.0	
Physically disabled		0.0079
No*	5.3 ± 7.8	
Yes	8.1 ± 9.3	
Permanently ill patient		0.0007
No*	6.0 ± 8.3	
Yes	3.8 ± 6.7	

“*” indicates the reference group.

**Table 6:** Percentage distribution of household food insecurity scores by basic needs.

Indicators of SDGs	Total (N = 869)		
	Food Security* (N = 476)	Food insecurity* (N = 393)	p-value
Healthcare Services Needs			0.0014
Met	45% (N = 214)	33% (N = 129)	
Somewhat met	32% (N = 154)	38% (N = 150)	
Not met at all	23% (N = 108)	29% (N = 114)	
Educational needs			< .0001
Met	62% (N = 294)	41% (N = 161)	
Somewhat met	26% (N = 122)	33% (N = 131)	
Not met at all	12% (N = 58)	26% (N = 101)	
Employment Needs			< .0001
Met	46% (N = 217)	18% (N = 71)	
Somewhat met	25% (N = 119)	47% (N = 185)	
Not met at all	29% (N = 139)	35% (N = 137)	
Financial Needs			< .0001
Met	43% (N = 205)	7% (N = 27)	
Somewhat met	48% (N = 229)	64% (N = 252)	
Not met at all	9% (N = 42)	29% (N = 114)	
Satisfactory family relationships			< .0001
Met	71% (N = 337)	37% (N = 145)	
Somewhat met	27% (N = 129)	39% (N = 154)	
Not met at all	2% (N = 10)	24% (N = 94)	
Water, Sanitation and Hygiene needs			0.0002
Met	32% (N = 153)	20% (N = 78)	
Somewhat met	34% (N = 162)	41% (N = 163)	
Not met at all	34% (N = 161)	38% (N = 151)	
Drinking Water needs			< .0001
Met	43% (N = 204)	30% (N = 118)	
Somewhat met	34% (N = 162)	36% (N = 142)	
Not met at all	23% (N = 110)	34% (N = 133)	
Electricity Needs			< .0001
Met	92% (N = 435)	64% (N = 252)	
Somewhat met	7% (N = 34)	16% (N = 64)	
Not met at all	1% (N = 6)	20% (N = 77)	
General Safety Needs			< .0001
Met	61% (N = 288)	40% (N = 159)	
Somewhat met	25% (N = 118)	29% (N = 114)	
Not met at all	15% (N = 70)	31% (N = 120)	
Woman Safety Needs			< .0001
Met	15% (N = 300)	44% (N = 174)	
Somewhat met	29% (N = 135)	24% (N = 92)	
Not met at all	8% (N = 40)	32% (N = 127)	
Child's Health and education need			< .0001
Met	64% (N = 306)	41% (N = 162)	
Somewhat met	29% (N = 140)	35% (N = 136)	
Not met at all	6% (N = 29)	24% (N = 95)	
Mentally ill			0.1058
Yes	3% (N = 14)	5% (N = 20)	
No	97% (N = 462)	95% (N = 373)	
Physically disabled			0.0354
Yes	5% (N = 25)	9% (N = 35)	
No	95% (N = 451)	91% (N = 358)	
Permanently ill patient			0.0207
Yes	26% (N = 126)	20% (N = 78)	
No	74% (N = 350)	80% (N = 315)	

“*” indicates variables with missing values (n = 38). Analysis was conducted using complete observations for the food insecurity variable (HFIAS).

Study participants reporting that their basic needs were met had significantly lower household food insecurity scores compared with those whose needs were not met at all (p<0.05). Household food insecurity scores were higher among study participants whose basic needs were somewhat met compared with those whose needs were met (Table 5). Among study participants reporting that their basic needs were met, food insecurity scores were highest for healthcare service needs (Mean = 3.9, SD = 6.5) and educational needs (Mean = 3.5, SD = 6.2), and were lowest for financial needs (Mean = 1.1, SD = 3.9). Having household members that were either mentally ill, physically disabled or permanently ill, was associated with higher food insecurity scores among the study participants (p<0.05) (Table 5). The highest burden of food insecurity was seen among study participants with mentally ill household members (Mean = 9.2, SD = 10).

### Predictors of household food insecurity

SDG-related predictors of food insecurity in the multivariable analysis included: highest level of education attained in the household (*SDG 4 Quality education) (p = 0*.*03)*, coverage of healthcare service needs (*SDG 3 Good health and well-being*) *(p = 0*.*0002)*, electricity needs (*SDG7 affordable and clean energy*) (p<0.0001), and employment needs (*SDG8 Decent and economic growth*) *(p = 0*.*003)* (Table 7).

**Table 7:** Multivariable analysis showing predictors of household food insecurity.

Variables	OR [95% CI]	p-value
Total number of household members	1.109 [1.027, 1.198]	0.0060
Highest level of education attained by family member		0.0334
No School*		
Incomplete school	0.967 [0.534, 1.753]	0.9001
High school diploma	0.898 [0.440, 1.833]	0.7392
Some college	1.752 [0.626, 4.908]	0.2869
Graduate/ Post-graduate	0.316 [0.121, 0.820]	0.0171
Number of working hours of the earning member*		0.0028
None*		
6 hours or less	0.224 [0.066, 0.762]	0.0156
7 to 8 hours	0.163 [0.052, 0.511]	0.0019
9 to 10 hours	0.237 [0.072, 0.776]	0.018
More than 10 hours	0.382 [0.113, 1.294]	0.1234
Healthcare Services Needs		0.0033
Met	2.640 [1.321, 5.276]	0.0066
Somewhat met	2.912 [1.562, 5.429]	0.0008
Not met at all*		
Financial Needs		< .0001
Met	0.161 [0.067, 0.385]	< .0001
Somewhat met	0.927 [0.473, 1.814]	0.8135
Not met at all*		
Satisfactory Family Relationships		0.0442
Met	0.331 [0.111, 0.986]	0.0474
Somewhat met	0.509 [0.176, 1.471]	0.2099
Not met at all*		
Employment Needs		0.0031
Met	1.134 [0.592, 2.172]	0.7261
Somewhat met	2.161 [1.266, 3.688]	0.0046
Not met at all*		
Electricity Needs		0.004
Met	0.138 [0.035, 0.542]	0.0049
Somewhat met	0.298 [0.073, 1.220]	0.0889
Not met at all*		
Women Safety Needs		< .0001
Met	0.192 [0.082, 0.446]	0.0001
Somewhat met	0.144 [0.064, 0.324]	< .0001
Not met at all*		
Physically disabled person in a household	2.299 [1.084, 4.876]	0.03

“*” indicates the reference group.

Having healthcare services needs that were partially or fully met were equally associated with higher odds of food insecurity (p<0.05). Failure to fully meet employment needs (OR = 2.161, 95% CI: 1.266, 3.688, p = 0.0046) was significantly associated with higher odds of food insecurity. However, having electricity needs that were met (OR = 0.138, 95% CI: 0.035, 0.542, p = 0.049), as well as financial needs that were met (OR = 0.161, 95% CI: 0.067, 0.385, p<0.0001) were significantly associated with a reduced odds of food insecurity (Table 7).

Household-level predictors of food insecurity included: the number of household members (OR = 1.109, 95% CI: 1.027, 1.198, p = 0.006), and the presence of physically disabled household members (OR = 2.299, 95% CI: 1.084, 4.876, p = 0.03). The highest level of education attained by family members was also significantly associated with food insecurity (p = 0.03). Study participants within households having at most a graduate degree were less likely to be food insecure (OR = 0.316, 95% CI: 0.121, 0.820, p = 0.0171). In addition, living in households where the earning members worked for up to 10 hours at most was significantly associated with reduced odds of food insecurity (OR = 0.237, 95% CI: 0.072, 0.776, p = 0.018) (Table 7).

## Discussion

Results showed that 43% (n = 393) of the study participants experienced food insecurity. More than half of them were females (73%, n = 285), had no schooling (51%, n = 202), and 48% (n = 190) were from households where most individuals had not completed high school. About one-third of the earning members in food insecure households worked for about 7 to 8 hours per day (34%, n = 128). Thirty-three percent of these households were located in the Northern Region of Delhi (n = 128). The prevalence of food insecurity was substantially higher in this study (43%) compared with other slum studies in New Delhi, India (30.6%) [8]. However, food insecurity prevalence was lower in the present study compared with similar developing countries such as Iran (59.1%) and the Philippines (65%) [10]. Correlates of food insecurity identified in our study were consistent with existing literature—showing that being female, having less than a high school education, working for fewer hours, and having large family sizes were predictors of food insecurity [10, 22].

Failure to fully meet employment needs remained significantly associated with an increased odds of food insecurity in the multivariable analysis. Likewise, study participants reporting that their employment needs were met, were also likely to be food insecure, although this was not significant in the multivariable analysis (OR = 1.134, 95% CI: 0.592, 2.172, p = 0.726). Prior studies have shown that employment-related food insecurity among the urban poor has a lot to do with the patterns of employment amongst urban poor slum dwellers [10, 22–24]. The finding that study participants with partially and fully met employment needs were likely to be food insecure suggests that being employed may not be fully reflective of income capacity. Household income has been identified as an important factor that determines household access to food [10]. This, in turn is dependent on access to remunerative employment [10]. While employment rates in India have rapidly grown in the past decade, the majority of this growth has been restricted to self-employment or informal sector employment. These forms of employment are characterized by low average earnings and are reflective of distress or limited access to better-wage employment, rather than expansion in productive employment opportunities [10]. Prior studies have also shown that being partially employed or unemployed are equally associated with food insecurity [25].

Study participants who reported having a physically disabled person in their household were twice as likely to be food insecure. This is consistent with prior findings that having a disabled household member increases the burden of food insecurity [26]. A study showed that households having children with disabilities were more likely to report food insecurity of any kind compared to other households [26].

Having healthcare services needs that were partially or fully met was equally associated with higher odds of food insecurity. Our study finding showing that having “met healthcare needs” was significantly associated with food insecurity is not consistent with existing literature. These findings could be attributed to the high cost of healthcare in urban settings, leading to high out-of-pocket costs which slum residents have to pay to receive healthcare services. In addition, the lack of federal subsidies by India’s Public Distribution System to un-notified slums might largely contribute to higher out-of-pocket costs. These factors could exacerbate food insecurity among slum dwellers in un-notified slum settings.

Study participants whose women’s safety needs were met were less likely to be food insecure. Women’s safety and security in slums have been linked to a variety of infrastructural elements. In particular, establishing drainage systems, septic tanks for improved sanitation, electricity and water supply connections for households are some of the infrastructural elements that have been shown to discourage the practice of open defaecation—a practice that creates insecurity among women [27]. According to prior research on slums in Delhi, India, “*In urban India women will reject substandard public or community latrines in favour of open defecation if they perceive the bodily harm or the risk of gender-based violence to be greater using the latrine*. *Women’s fear and stress then*, *is not a problem with sanitation*, *but with social inequalities that put women at risk of gender-based violence*” [28].

Having financial needs that were met—was protective of food insecurity in the present study. This finding is consistent with similar research in Indian slums showing that families with higher household debt (used as a proxy for financial capacity) were more likely to be food insecure [10]. An increase in household food insecurity status was related to a decline in socio-economic status [10]. According to a report on the state of food security in Urban India, issues related to access and utilization (such as subsidized food costs provided by India’s public distribution system mostly to notified slums) are more predictive of food security, in comparison to food availability in stores [29]. The urban poor are more likely to spend 60% more of their earnings on food than the rural poor [10].

Correlates of food security identified in our study: financial needs, satisfactory family relationships, electricity needs and women’s safety needs, were consistent with prior studies in varied settings [30]. Results of this study do not demonstrate any causal or quasi-causal claims, but simply identify predictors of food insecurity in un-notified Indian slums. Studies utilizing longitudinal data are needed to investigate the possible causal relationships between the SDG- related predictors from this study and food insecurity in urban slums, while accounting for other possible determinants such as seasonal variations in food access, food availability and other environmental variables. Food insecurity is multidimensional and multifactorial in nature. Identifying risk factors associated with food insecurity is not only essential for gaining a complete understanding of the situation but also for designing interventions needed to address them.

Long-term interventions and multifaceted initiatives are needed to positively impact and prevent food insecurity. These solutions should include connecting food insecure households especially in un-notified slums to existing social services such as India’s Public Distribution System, while addressing the underlying causes of food insecurity, such as unemployment, underemployment, limited household resources/assets, unstable housing, poor health, low education, and poverty. Further research is also needed to explore the role of healthcare costs in relation to food insecurity among residents in un-notified slum settings. Multisector partnerships are needed to develop the necessary mechanisms and infrastructure that address educational, employment, health service, women safety, electricity and family relationship needs in slum settings, with the goal of reducing food insecurity. Such infrastructure may include the establishment of improved sanitation, electricity and water supply connections that have been shown to improve women’s safety in urban slums.

## Supporting information

S1 Appendix HFIAS indicator guide. This is the household food insecurity access scale.
(PDF)
