# Supplementary material for: Burden of household food insecurity in urban slum settings
Source: PLoS One. 2019 Apr 2;14(4):e0214461. doi: 10.1371/journal.pone.0214461 (PMC6445475; doi:10.1371/journal.pone.0214461)
Supplement: S1 Appendix — This is the household food insecurity access scale. (PDF) [file pone.0214461.s001.pdf]

FANTA III

FOOD AND NUTRITION TECHNICAL ASSISTANCE

USAID FROM THE AMERICAN PEOPLE

Household Food Insecurity Access Scale (HFIAS) for Measurement of Food Access: Indicator Guide

VERSION 3

August 2007

Jennifer Coates
Anne Swindale
Paula Bilinsky

FANTA
FHI 360
1825 Connecticut Ave., NW
Washington, DC 20009-5721
Tel: 202-884-8000 Fax: 202-884-8432
fantamail@fhi360.org www.fantaproject.org

fhi 360 THE SCIENCE OF IMPROVING LIVES

FANTA III

FOOD AND NUTRITION TECHNICAL ASSISTANCE

USAID FROM THE AMERICAN PEOPLE

Household Food Insecurity Access Scale (HFIAS) for Measurement of Food Access: Indicator Guide

VERSION 3

Jennifer Coates
Anne Swindale
Paula Bilinsky

August 2007

Food and Nutrition Technical Assistance III Project (FANTA)
FHI 360   1825 Connecticut Avenue, NW   Washington, DC 20009-5721
T 202-884-8000   **F** 202-884-8432   fantamail@fhi360.org   www.fantaproject.org

This publication was made possible by the generous support of the American people through the support of the U.S. Agency for International Development (USAID) Bureau for Global Health, Office of Health, Infectious Diseases, and Nutrition, under terms of Cooperative Agreement No. HRN-A-00-98-00046-00, through the Food and Nutrition Technical Assistance Project (FANTA), managed by FHI 360.

The contents are the responsibility of FHI 360 and do not necessarily reflect the views of USAID or the United States Government.

August 2007

Recommended citation:

Coates, Jennifer, Anne Swindale and Paula Bilinsky. 2007. *Household Food Insecurity Access Scale (HFIAS) for Measurement of Household Food Access: Indicator Guide (v. 3).* Washington, D.C.: FHI 360/FANTA.

Copies of the publication can be obtained from:

Food and Nutrition Technical Assistance Project (FANTA)
FHI 360
1825 Connecticut Avenue, NW
Washington, D.C. 20009-5721
T: 202-884-8000
F: 202-884-8432
fantamail@fhi360.org
www.fantaproject.org

Table of Contents

Acknowledgments i
1. Background 1
2. Adapting the Questionnaire 5
2.1. Overview of Questionnaire 5
2.2. Step 1: Review with Key Informants 6
2.3. Step 2: Refining the Questionnaire 7
3. Interviewer Instructions 9
3.1. Organization of the HFIAS Questionnaire 9
3.2. Asking Questions and Recording Answers 10
3.3. Instructions for Individual Questions 11
4. Questionnaire Format 13
5. Indicator Tabulation Plan 16
5.1 Household Food Insecurity Access-related Conditions 16
5.2 Household Food Insecurity Access-related Domains 17
5.3 Household Food Insecurity Access Scale Score 17
5.4 Household Food Insecurity Access Prevalence 18
References 21
Appendix 1: Key Informant Interview Guide 23
Endnotes 28

Acknowledgments

This guide could not have been completed without the intellectual and technical contributions of Edward Frongillo, Cornell University and Beatrice Lorge Rogers, Tufts University. The guide reflects very useful and practical feedback on earlier drafts from staff from the following institutions: Africare, American Red Cross, Catholic Relief Services, Food and Agriculture Organization of the United Nations (European Commission Programme on Food Security), Food for the Hungry International, Freedom from Hunger, Land O'Lakes International Development, National Center for Health Statistics, Save the Children-US, World Vision United States, U.S. Agency for International Development (USAID), and U.S. Department of Agriculture's Economic Research Service. We have also greatly benefited from the vision and support of Eunyong Chung at USAID throughout the process of developing the Household Food Insecurity Access Scale.

In Version 3 of the guide, the HFIAS questions have been refined to address the recommendations of the Nutrition and Consumer Protection Division, Food and Agriculture Organization of the United Nations (FAO), which carried out HFIAS adaptation work in multiple countries under the EC/FAO Food Security Information for Action Programme. We thank the Nutrition and Consumer Protection Division of FAO for its partnership with FANTA in work related to the HFIAS.

1. Background

Food security is defined as a state in which "all people at all times have both physical and economic access to sufficient food to meet their dietary needs for a productive and healthy life" (USAID, 1992).[i] Because it is a complex, multidimensional concept, measuring food insecurity has been an ongoing challenge to researchers and practitioners alike. Until very recently, most household-level measures of food access, such as income and caloric adequacy, have been technically difficult, data-intensive, and costly to collect.

USAID Title II and Child Survival and Health Grant programs require relatively simple, but methodologically rigorous, indicators of the access component of household food insecurity (hereafter referred to as household food insecurity (access)) that can be used to guide, monitor and evaluate program interventions. Over the past several years, USAID's Food and Nutrition Technical Assistance (FANTA) project has supported a series of research initiatives to explore and test different options for meeting this need.

This document is a guide for implementing one such option, the Household Food Insecurity Access Scale (HFIAS), which is an adaptation of the approach used to estimate the prevalence of food insecurity in the United States (U.S.) annually. The method is based on the idea that the experience of food insecurity (access) causes predictable reactions and responses that can be captured and quantified through a survey and summarized in a scale. Qualitative research with low-income households in the U.S. provided insight into the following ways that households experience food insecurity (access) (Radimer et al., 1990, Radimer et al., 1992, Wehler et al., 1992, Hamilton, 1997):

- Feelings of *uncertainty* or *anxiety* over food (situation, resources, or supply);
- Perceptions that food is of insufficient *quantity* (for adults and children);
- Perceptions that food is of insufficient *quality* (includes aspects of dietary diversity, nutritional adequacy, preference);
- Reported *reductions* of food intake (for adults and children);
- Reported *consequences* of reduced food intake (for adults and children); and
- Feelings of *shame* for resorting to socially unacceptable means to obtain food resources.[ii]

The eighteen-question U.S. Household Food Security Survey Module (US HFSSM) asks respondents to describe behaviors and attitudes that relate to these various aspects, also called 'domains', of the food insecurity experience (Hamilton et al., 1997). For example, a question relating to perceptions of insufficient *quantity* asks whether any adults had to eat less than they thought they should. The *uncertainty*-related questions include one about whether the respondent worried that the household's food would run out. Responses to the US HFSSM are summarized in a scale to provide a continuous indicator of the degree of a household's food insecurity. Cut-off points on the scale enable categorical classification of whether households are food secure or not. These data are used to monitor food assistance programs and to report on national prevalence of household food insecurity.

Recent field validation studies of this approach to measuring food insecurity (access) more directly, by constructing measures based on households' experience of the problem, have demonstrated the feasibility and usefulness of the approach in very different, developing country contexts (Webb et al., 2002, Coates et al., 2003, Frongillo and Nanama, 2003). The measures constructed were strongly correlated with common indicators of poverty and food consumption as well as with indicators currently used by Private

Voluntary Organizations (PVOs) to monitor their food security-related activities. They were also sensitive to changes in the households' situation over time, making them valid and useful for assessing program impact. There are other studies where US HFSSM questions have been translated, with some adaptation, to developing country settings and found to be correlated with poverty and food consumption indicators (Melgar-Quinonez, 2004, Perez-Escamilla et al., 2004). Furthermore, based on a review of evidence from 22 different scale applications, a paper examining commonalities in the experience and expression of food insecurity (access) across cultures identified four domains and several sub-domains of food insecurity (access) that appear to be universal across different countries and cultures. The paper recommended that questions related to these domains be used as the basis of future food insecurity (access) scale measures (Coates, 2005).

Based on this growing body of evidence, FANTA and its partners have identified a set of questions (see Table 1, Household Food Insecurity Access Scale Generic Questions) that have been used in several countries and appear to distinguish the food secure from the insecure households across different cultural contexts.[iii] These questions represent apparently universal domains[iv] of the household food insecurity (access) experience and can be used to assign households and populations along a continuum of severity, from food secure to severely food insecure. The information generated by the HFIAS can be used to assess the prevalence of household food insecurity (access) (e.g., for geographic targeting) and to detect changes in the household food insecurity (access) situation of a population over time (e.g., for monitoring and evaluation). The questions can be added to a standard baseline and final evaluation survey. When using the scale to determine impact, it is important to follow the standard sampling methods commonly used in Title II evaluations. A detailed discussion of sampling can be found at: http://www.fantaproject.org/publications/sampling.shtml.

If assessing the change in the household food insecurity (access) situation between two or more years, it is important to administer the survey at the same time of year each time. The most appropriate time of year should be determined based on the intended use of the scale. When using the scale to determine impact of a food security program, it is preferable to administer the survey during or directly after the worst of the 'lean season', because the greatest number of households is likely to be affected by food insecurity (access) at this time. This height of the lean season, however, may not be best if the scale is being used for geographical targeting, because the program may not be able to differentiate among those who are severely food insecure during many months of the year and those who are food insecure only during the lean season. This may be important if the program is attempting to target areas with the greatest number of chronically food insecure households.

The intent of this guide is to provide a means for food security programs to easily measure the impact of their programs on the <u>access</u> component of household food insecurity. Understanding and measuring the impact of programming on the <u>utilization</u> component of food insecurity is equally important, but is better accomplished using other measurement tools, such as anthropometric indicators. One aspect of utilization is the question of nutritional quality. In the context of the HFIAS, food quality questions do not refer directly to nutritional quality. Rather these questions attempt to capture the household's perception of changes to the quality of their diet regardless of the diet's objective nutritional composition (e.g., households may perceive that a change from rice to corn has caused a decline in the quality of their diet when the nutritional quality has not in fact changed significantly).

Efforts to measure food insecurity (access) have sometimes relied in part on an index of coping strategies. In earlier versions of the HFIAS, questions about a household's strategies to augment its resource base, such as taking a loan, were included in the scale along with questions about consumption-related coping strategies that ask about reductions or redistribution of food within the household, such as skipping meals

or eating less preferred foods. Further research and discussion has led FANTA to conclude that the former type of coping strategies (to augment the household resource base) should be excluded from the HFIAS. The reasons for this decision are as follows:

In order to construct an accurate scale, all the questions in the scale must reflect a single statistical dimension (unidimensionality), even if the phenomenon (in our case, food insecurity (access)) is multidimensional. Statistical models, such as the Rasch model used to develop the US HFSSM, showed that the questions about strategies to augment the resource base represent a distinct statistical dimension of household food insecurity (access) from the dimension measured by the domains in the HFIAS.

Questions about strategies to augment the resource base are subject to household supply and access constraints – that is, not all coping strategies are accessible or available to all families (e.g., taking a loan is not an option for extremely food insecure households to whom even informal moneylenders will not lend). Responses to these questions are therefore misleading because a negative response does not necessarily indicate that the household is food secure. For example, a very food secure family who did not need a loan and a family who could not get a loan would both respond negatively to a question about getting a loan, even though the latter is much more food insecure than the former.

The types of resource augmentation coping strategies that households resort to and the level of severity they indicate vary widely across cultures and countries, making it very difficult to identify a universally relevant set of resource augmentation questions.

Resource augmentation coping strategies are important to consider, however, in gaining a more detailed picture of the experience of food insecurity (access) in any particular context. Households that resort to unsustainable coping strategies, such as selling productive assets or taking high interest loans, represent a crucial area of concern for those working with the most food insecure populations. These household strategies, along with behaviors such as migration or begging, indicate the nature of the household's vulnerability. An examination of common resource augmentation coping strategies and their impact on food insecure households should be part of any program's initial food security assessment. Such coping strategies may represent areas that are amenable to program focus in order to increase household resiliency and as such are important to monitor as households' food security status changes.

Earlier versions of this guide also included the following question relating to the psychological effects, like feelings of shame, that result from having to use socially unacceptable strategies to get food: "Did you or any household member have to do something that made you feel ashamed because there was not enough food?" Though a cross-cultural review of ethnographic research on the experience of access-constrained food insecurity had concluded that this domain is a relevant aspect of the experience in many cultures, few studies have tried to ask questions about "shame from socially unacceptable strategies" in a survey. Those that did ask such questions sometimes found that the shameful or socially unacceptable actions and feelings were very sensitive issues and that it was difficult to elicit an accurate response. FANTA concluded that not enough field-based success existed for a 'generic' question to be included in the HFIAS questionnaire, so the question has been dropped from this revised version of the HFIAS. Further work is needed in order to determine the feasibility, and most appropriate way, of including the shame/social unacceptability dimension in a standardized HFIAS.

The rest of the guide is presented as follows: Section 2. *Adapting the Questionnaire and Probing* lists the generic questions and describes a two-step process to adapt the model questionnaire; Section 3. *Interviewer Instructions* provides specific instructions to the interviewers; Section 4. presents the *Model Questionnaire*; and Section 5. *Indicator Tabulation Plan* describes how the questions can be tabulated to make indicators and provides recommendations for their use and interpretation.[v]

Table 1: Household Food Insecurity Access Scale (HFIAS) Generic Questions

Each of the questions in the following table is asked with a recall period of four weeks (30 days). The respondent is first asked an occurrence question – that is, whether the condition in the question happened at all in the past four weeks (yes or no). If the respondent answers "yes" to an occurrence question, a frequency-of-occurrence question is asked to determine whether the condition happened rarely (once or twice), sometimes (three to ten times) or often (more than ten times) in the past four weeks.

Example:

1. In the past four weeks, did you worry that your household would not have enough food?

   0 = No (skip to Q2)

   1 = Yes

1.a. How often did this happen?

   1 = Rarely (once or twice in the past four weeks)

   2 = Sometimes (three to ten times in the past four weeks)

   3 = Often (more than ten times in the past four weeks)

No.	Occurrence Questions
1.	In the past four weeks, did you worry that your household would not have enough food?
2.	In the past four weeks, were you or any household member not able to eat the kinds of foods you preferred because of a lack of resources?
3.	In the past four weeks, did you or any household member have to eat a limited variety of foods due to a lack of resources?
4.	In the past four weeks, did you or any household member have to eat some foods that you really did not want to eat because of a lack of resources to obtain other types of food?
5.	In the past four weeks, did you or any household member have to eat a smaller meal than you felt you needed because there was not enough food?
6.	In the past four weeks, did you or any household member have to eat fewer meals in a day because there was not enough food?
7.	In the past four weeks, was there ever no food to eat of any kind in your household because of lack of resources to get food?
8.	In the past four weeks, did you or any household member go to sleep at night hungry because there was not enough food?
9.	In the past four weeks, did you or any household member go a whole day and night without eating anything because there was not enough food?

2. Adapting the Questionnaire

2.1. Overview of Questionnaire

The recommended questionnaire format for the HFIAS can be found in Section 4. The questionnaire consists of nine occurrence questions that represent a generally increasing level of severity of food insecurity (access), and nine "frequency-of-occurrence" questions that are asked as a follow-up to each occurrence question to determine how often the condition occurred. The frequency-of-occurrence question is skipped if the respondent reports that the condition described in the corresponding occurrence question was not experienced in the previous four weeks (30 days). Some of the nine occurrence questions inquire about the respondents' *perceptions* of food vulnerability or stress (e.g., did you worry that your household would not have enough food?) and others ask about the respondents' *behavioral responses* to insecurity (e.g., did you or any household member have to eat fewer meals in a day because there was not enough food?). The questions address the situation of all household members and do not distinguish adults from children or adolescents.[vi] All of the occurrence questions ask whether the respondent or other household members either felt a certain way or performed a particular behavior over the previous four weeks.[vii]

The HFIAS occurrence questions relate to three different domains of food insecurity (access) found to be common to the cultures examined in a cross-country literature review (FANTA 2004, Coates, 2004).[viii] The generic occurrence questions, grouped by domain, are:

1. Anxiety and uncertainty about the household food supply:
   - Did you worry that your household would not have enough food?

2. Insufficient Quality (includes variety and preferences of the type of food):
   - Were you or any household member not able to eat the kinds of foods you preferred because of a lack of resources?
   - Did you or any household member have to eat a limited variety of foods due to a lack of resources?
   - Did you or any household member have to eat some foods that you really did not want to eat because of a lack of resources to obtain other types of food?

3. Insufficient food intake and its physical consequences:
   - Did you or any household member have to eat a smaller meal than you felt you needed because there was not enough food?
   - Did you or any household member have to eat fewer meals in a day because there was not enough food?
   - Was there ever no food to eat of any kind in your household because of a lack of resources to get food?
   - Did you or any household member go to sleep at night hungry because there was not enough food?
   - Did you or any household member go a whole day and night without eating anything because there was not enough food?

The questionnaire should be asked in its entirety, however, the enumerator should follow the embedded skip rules to avoid asking frequency-of-occurrence questions when they are not applicable. Project staff should avoid picking and choosing only certain questions. Though users may want to report the results of individual questions alongside other indicators (see Section 5), research has shown that the complete set of questions does a better job of distinguishing the household food insecurity (access) level than any question on its own.

The questions in the model questionnaire are worded to be as universally relevant as possible. Certain questions contain *phrases*, however, that may need to be adapted to the local context to ensure that respondents know their meaning. Some questions require that the interviewer read a locally appropriate definition (e.g., of 'household') the first time these words are used in a question. Finally, certain questions may require that the interviewer provide locally relevant *examples* when the respondent requires further prompting.

In order to adapt the phrases, definitions, and examples to the local context and to ensure that questions are understood appropriately, they should be reviewed with a group of key informants and then refined with a small group of respondents before the pre-test. A detailed description of the process of discussing the questions with key informants is provided in Appendix 1. These two steps are described briefly below:

2.2. Step 1: Review with Key Informants

As a first step, gather a few key informants who are familiar with the conditions and experiences of household food insecurity (access) in the areas where the survey will be conducted. These key informants could be PVO staff members, government officials, academics, prominent community members, or other knowledgeable individuals. It should be explained to the key informants that they are being consulted to ensure that the food insecurity (access) questions are understandable in their country or culture. They should also be given the option to participate or not, and should be informed that they can choose to leave or refuse to answer a question at any time. Where possible, the key informants should be consulted as a group, so that any discrepancies in their suggestions can be clarified at the same time.

The person conducting the key informant interviews (the "Interviewer") should follow the Key Informant Interview Guide, presented in Appendix 1. The Interviewer should read each question to the key informant and then read the probes listed below that question. For instance, the Interviewer should read:

"Q1: Did you worry that your [household] would not have enough food?"

Then the Interviewer should read the following probe:

We would like to add a culture-specific definition of "household." For instance, in some cultures "household" might be defined as "people who live together and share food from a common pot." Can you tell us how people here commonly describe a household?

The word or phrases that the key informants should focus on are written in bold in the Key Informant Interview Guide.

After the informant has the chance to respond, and once the Interviewer is satisfied that he or she has enough information to adapt the question appropriately, then the Interviewer should move on to each subsequent question in the Key Informant Interview Guide, using the same procedure. All of the discussions with the informants should be recorded by a note-taker.

At the conclusion of the key informant interviews, the key informants' suggestions for adapting phrases and examples should be incorporated into the questionnaire. Included in the Key Informant Interview Guide in Appendix 1 are text boxes with examples of how each question might look after the information from key informants has been integrated. The final product of this step should be a draft questionnaire, with locally relevant phrases and examples where necessary, that can be tested with a group of respondents in Step 2.

2.3. Step 2: Refining the Questionnaire

The second step in preparing the questionnaire is to ensure that the questions are understood by respondents as they are intended. This step, which is very important in any survey context, enables further refinement of the questions and examples based on insights into how the questions are actually being interpreted.

Identify 8-10 individuals that are representative of the survey population (but who are not part of the survey sample). As with the key informants, these individuals should also be informed of the option to participate or not, and should be informed that they can choose to leave or refuse to answer a question at any time.

For this step, the discussions are best done with one respondent at a time. First, the Interviewer should read the question, including any suggested rephrasing or examples incorporated after the key informant session. After the respondent has a chance to provide a response, the Interviewer should begin to explore the respondent's own understanding of the question and its meaning. Tips for doing so are included in Table 2. A note-taker should record these discussions. Once all of the respondents have provided their input, the notes from all of these discussions should be pooled and examined. Based on respondent feedback, particular phrases, definitions, words, or examples that were unclear should be reworded accordingly. Remember, the goal is to retain the original meaning of the question while making the meaning clearer to respondents where necessary. The final product of this step should be an improved draft questionnaire that is ready to be pre-tested in the field.

Table 2. Example Probes for Use in Refining Questions with Respondents

Comprehension/ interpretation probes	When I asked you about..., what were you thinking about?<br>Can you tell me in your own words what this question means?<br>In thinking about..., what comes to mind?<br>*Examples:*<br>What does the phrase "eat a limited variety of foods" mean to you?<br>In your own words, can you tell me what "not enough food" means?
Paraphrase	Can you repeat the question in your own words?
Recall probe	How did you remember? For example, how did you remember that another household member went to sleep at night hungry because there was not enough food?
Specific probe	Why do you think that? For example, why do you consider those foods as ones you really did not want to eat?
General probes	How did you arrive at that answer?<br>How hard was that to answer?<br>I noticed that you hesitated before you answered -- what were you thinking about?

Adapted from Frongillo et al., 2004

3. Interviewer Instructions

3.1. Organization of the HFIAS Questionnaire

The HFIAS consists of two types of related questions. The first question type is called an occurrence question. There are nine occurrence questions that ask whether a specific condition associated with the experience of food insecurity *ever* occurred during the previous four weeks (30 days). Each severity question is followed by a frequency-of-occurrence question, which asks *how often* a reported condition occurred during the previous four weeks.

Each occurrence question consists of the stem (timeframe for recall), the body of the question (refers to a specific behavior or attitude), and two response options (0 = no, 1 = yes). There is also a 'skip code' next to each "no" response option. This code instructs the enumerator to skip the related frequency-of-occurrence follow-up question whenever the respondent answers "no" to an occurrence question.

Each HFIAS frequency-of-occurrence question asks the respondent how often the condition reported in the previous occurrence question happened in the previous four weeks. There are three response options representing a range of frequencies (1 = rarely, 2 = sometimes, 3 = often). Table 3 illustrates these different question components and can be referred to in using these instructions.

Table 3. Structure of Questions

	**Occurrence Question**
Body	In the past four weeks, did you worry that your household would not have enough food?
Response Options	0=No (Skip to …)<br>1=Yes
	**Frequency-of-occurrence Question**
Body	How often did this happen?
Response Options	1=Rarely (once or twice in the past four weeks)<br>2=Sometimes (three to ten times in the past four weeks)<br>3=Often (more than ten times in the past four weeks)

3.2. Asking Questions and Recording Answers

The questions should be directed to the person in the household who is most involved with the food preparation and meals.[ix] Most of the questions require the respondent to answer on behalf of the household and all its members.

There are two terms used throughout the questionnaire that are highly context specific: “household” and “lack of resources.” Context-specific definitions for these terms should have been developed during the questionnaire adaptation phase and added to the questionnaire. The definitions for these terms should be read by the interviewer the first time they are used in a question. These definitions and the questions themselves of should be read just as they are written on the questionnaire.

Below is an example of an occurrence question with an interviewer-provided definition. The entire thing should be read by the enumerator:

Q1: In the past four weeks, did you worry that your household would not have enough food?

By “household” we mean those of you that sleep under the same roof and take meals together at least four days a week.

If the respondent does not understand the question, then the interviewer may prompt the respondent by reading any examples or contextual clarifications that were discussed during training. These interviewer-provided examples are written in *italics* below the question itself. For example, a question with an interviewer-provided example might appear as follows:

Q4: In the past four weeks, did you or any household member have to eat some foods that you really did not want to eat because of a lack of resources?

Interviewer-provided example: “A food you really did not want to eat” might include wheat porridge, wild taro root, etc.

Although there are pre-coded response options, the interviewer should not read these options aloud each time but rather allow the respondent to answer in his or her own words. The interviewer will select the most appropriate response option based on the respondent’s reply. For instance if, after asking an occurrence question, the respondent says “no” but adds that it only happened a few times, then the correct code is ‘1’ (yes). The frequency-of-occurrence question should then be asked. If the respondent describes a frequency that would translate to “three to ten times” in the past four weeks, the correct response selection for the frequency-of-occurrence question is “sometimes”, and the correct code is ‘2’. If the respondent has difficulty replying then the interviewer can encourage a response by listing the set of options again.

The box below illustrates the example described, above:

No	Question	Response Options	Code
Q7.	In the past four weeks, was there ever no food to eat of any kind in your household because of lack of resources to get food?<br><br>Respondent Answer: No. Well, just a few times.	0 = No (skip to Q.8)<br><br>1 = Yes	1
Q.7.a.	How often did this happen in the past four weeks?<br><br>Respondent Answer: four times	1=Rarely (once or twice in the past four weeks)<br><br>2 = Sometimes (three to ten times in the past four weeks)<br><br>3 = Often (more than ten times in the past four weeks)	2

After completing the questionnaire and before leaving the household, interviewers should check over the questionnaires to ensure that all questions have been asked and that the responses are complete and legible. They may wish to write notes in the margins next to any unusual responses or stories that emerged in relation to a particular question. Such notes can help later on in interpreting the data from the entire sample. The administration of the questionnaire requires approximately 15 minutes per household.

3.3. Instructions for Individual Questions

Q1: Worry about food

This question asks the respondent to report their personal experience with uncertainty and anxiety about acquiring food during the previous month. The interviewer should also read the definition of a "household" that was developed during the preparation of the questionnaire. Mention that this definition of household applies to all the questions with that term.

Q2: Unable to eat preferred foods

One domain of food insecurity (access) is having limited choices in the type of food that a household eats. This question asks whether any household member was not able to eat according to their preference due to a lack of resources. Preference can refer to the form of a particular food (i.e., whole rice vs. broken rice), type of staple (i.e., millet vs. corn) or a high quality food (i.e., a piece of meat or fish). Preferred foods may or may not be nutritionally high quality. The interviewer should also read the definition of a "lack of resources." Mention that this definition of household applies to all the questions with that term. The respondent needs to answer on behalf of all household members

Q3: Eat just a few kinds of foods

This question asks about dietary choices related to variety – i.e., whether the household had to eat an undesired monotonous diet (little diversity in the different types of foods consumed). The interviewer should read the description of what a monotonous diet might be. The respondent needs to answer on behalf of all household members.

Q4: Eat foods they really do not want eat

This question, which also captures the dimension of limited choices, asks whether any household member had to eat food that they found socially or personally undesirable due to a lack of resources. Often these are foods or food preparations that are consumed only under hardship. Different people may consider different foods to be undesirable, so it is best not to provide examples here at first. The respondent needs to answer on behalf of all household members, according to his or her own perception of the types of food household members ate during the previous four weeks. If more encouragement is required, the interviewer may give some examples using any examples included in the questionnaire and reviewed during training. For all questions, it is important to remind respondents that the examples are not an exhaustive list.

Q5: Eat a smaller meal

This question asks whether the respondent felt that the amount of food (any kind of food, not just the staple food) that any household member ate in any meal during the past four weeks was smaller than they felt they needed due to a lack of resources. The respondent should answer according to his or her perception of what constitutes enough food for the needs of the household members. The respondent needs to answer on behalf of all household members.

Q6: Eat fewer meals in a day

This question asks whether any household member, due to lack of food, had to eat fewer meals than the number typically eaten in the food secure households in their area. The respondent needs to answer on behalf of all household members.

Q7: No food of any kind in the household

This question asks about a situation in which the household has no food to eat of any kind in the home. This describes a situation where food was not available to household members through the households' usual means (e.g., through purchase, from the garden or field, from storage, etc.).

Q8: Go to sleep hungry

This question asks whether the respondent felt hungry at bedtime because of lack of food or whether the respondent was aware of other household members who were hungry at bedtime because of lack of food. The respondent needs to answer on behalf of all household members.

Q9: Go a whole day and night without eating

This question asks whether any household member did not eat from the time they awoke in the morning to the time they awoke the next morning due to lack of food. The respondent needs to answer on behalf of all household members.

4. Questionnaire Format

Table 4. Household Food Insecurity Access Scale (HFIAS) Measurement Tool

No	Question	Response Options	CODE
1.	In the past four weeks, did you worry that your household would not have enough food?	0 = No (skip to Q2)<br>1=Yes	....|___|
1.a	How often did this happen?	1 = Rarely (once or twice in the past four weeks)<br>2 = Sometimes (three to ten times in the past four weeks)<br>3 = Often (more than ten times in the past four weeks)	....|___|
2.	In the past four weeks, were you or any household member not able to eat the kinds of foods you preferred because of a lack of resources?	0 = No (skip to Q3)<br>1=Yes	....|___|
2.a	How often did this happen?	1 = Rarely (once or twice in the past four weeks)<br>2 = Sometimes (three to ten times in the past four weeks)<br>3 = Often (more than ten times in the past four weeks)	....|___|
3.	In the past four weeks, did you or any household member have to eat a limited variety of foods due to a lack of resources?	0 = No (skip to Q4)<br>1 = Yes	....|___|
3.a	How often did this happen?	1 = Rarely (once or twice in the past four weeks)<br>2 = Sometimes (three to ten times in the past four weeks)<br>3 = Often (more than ten times in the past four weeks)	....|___|
4.	In the past four weeks, did you or any household member have to eat some foods that you really did not want to eat because of a lack of resources to obtain other types of food?	0 = No (skip to Q5)<br>1 = Yes	....|___|
4.a	How often did this happen?	1 = Rarely (once or twice in the past four weeks)<br>2 = Sometimes (three to ten times in the past four weeks)<br>3 = Often (more than ten times in the past four weeks)	....|___|

No	Question	Response Options	CODE
5.	In the past four weeks, did you or any household member have to eat a smaller meal than you felt you needed because there was not enough food?	0 = No (skip to Q6)<br>1 = Yes	....|___|
5.a	How often did this happen?	1 = Rarely (once or twice in the past four weeks)<br>2 = Sometimes (three to ten times in the past four weeks)<br>3 = Often (more than ten times in the past four weeks)	....|___|
6.	In the past four weeks, did you or any other household member have to eat fewer meals in a day because there was not enough food?	0 = No (skip to Q7)<br>1 = Yes	....|___|
6.a	How often did this happen?	1 = Rarely (once or twice in the past four weeks)<br>2 = Sometimes (three to ten times in the past four weeks)<br>3 = Often (more than ten times in the past four weeks)	....|___|
7.	In the past four weeks, was there ever no food to eat of any kind in your household because of lack of resources to get food?	0 = No (skip to Q8)<br>1 = Yes	....|___|
7.a	How often did this happen?	1 = Rarely (once or twice in the past four weeks)<br>2 = Sometimes (three to ten times in the past four weeks)<br>3 = Often (more than ten times in the past four weeks)	....|___|
8.	In the past four weeks, did you or any household member go to sleep at night hungry because there was not enough food?	0 = No (skip to Q9)<br>1 = Yes	....|___|
8.a	How often did this happen?	1 = Rarely (once or twice in the past four weeks)<br>2 = Sometimes (three to ten times in the past four weeks)<br>3 = Often (more than ten times in the past four weeks)	....|___|
9.	In the past four weeks, did you or any household member go a whole day and night without eating anything because there was not enough food?	0 = No (questionnaire is finished)<br>1 = Yes	....|___|

No	Question	Response Options	CODE
9.a	How often did this happen?	1 = Rarely (once or twice in the past four weeks)<br>2 = Sometimes (three to ten times in the past four weeks)<br>3 = Often (more than ten times in the past four weeks)	....|___|

5. Indicator Tabulation Plan

This section provides guidance on analyzing the data to create HFIAS indicators. It assumes that these questions will be part of a population-based survey instrument and will be applied to all the households in the sample.

The HFIAS module yields information on food insecurity (access) at the household level. Four types of indicators can be calculated to help understand the characteristics of and changes in household food insecurity (access) in the surveyed population. These indicators provide summary information on:

Household Food Insecurity Access-related ***Conditions***

Household Food Insecurity Access-related ***Domains***

Household Food Insecurity Access ***Scale Score***

Household Food Insecurity Access ***Prevalence***

The responses from the household food insecurity (access) measure should be entered into a database, spreadsheet, or statistical software like EpiInfo or SPSS. Computer tabulation is recommended for these indicators, though if necessary the data may also be tabulated by hand.

5.1 Household Food Insecurity Access-related Conditions

These indicators provide specific, disaggregated information about the behaviors and perceptions of the surveyed households. For example, if a program is providing assistance in growing staple crops and improved storage facilities, it might be useful to understand what percent of households had run out of food. The indicators present the percent of households that responded affirmatively to each question, regardless of the frequency of the experience. Thus they measure the percent of households experiencing the condition at any level of severity. Each indicator can be further disaggregated to examine the frequency of experience of the condition across the surveyed households.

**Household Food Insecurity Access-related Conditions**<br><br>Households experiencing condition at any time during the recall period.	Percent of households that responded, “yes” to a specific occurrence question. For example: “Percent of households that ran out of food.”<br><br>Example:<br><br>$$\frac{\text{Number of households with response = 1 to Q7}}{\text{Total number of households responding to Q7}} \times 100$$
Households experiencing condition at a given frequency	Percent of households that responded “often” to a specific frequency-of-occurrence question. For example: “Percent of households that ran out of food often.”<br><br>Example:<br><br>$$\frac{\text{Number of households with response = 3 to Q7a}}{\text{Total number of households responding to Q7}} \times 100$$

5.2 Household Food Insecurity Access-related Domains

These indicators provide summary information on the prevalence of households experiencing one or more behaviors in each of the three domains reflected in the HFIAS—Anxiety and uncertainty, Insufficient Quality, and Insufficient food intake and its physical consequences.

**Household Food Insecurity Access-related Domains**<br><br>Households experiencing any of the conditions at any level of severity in each domain	Percent of households that responded "yes" to any of the conditions in a specific domain. For example: "Percent of households with insufficient food quality."<br><br>Example:<br><br>Number of households with response = 1 to Q2 OR 1 to Q3 OR 1 to Q4<br>———————————————— X 100<br>Total number of households responding to Q2 OR Q3 OR Q4

5.3 Household Food Insecurity Access Scale Score

The HFIAS score is a continuous measure of the degree of food insecurity (access) in the household in the past four weeks (30 days). First, a HFIAS score ***variable*** is calculated for each household by summing the codes for each frequency-of-occurrence question. Before summing the frequency-of-occurrence codes, the data analyst should code frequency-of-occurrence as 0 for all cases where the answer to the corresponding occurrence question was "no" (i.e., if Q1=0 then Q1a=0, if Q2=0 then Q2a =0, etc.). The maximum score for a household is 27 (the household response to all nine frequency-of-occurrence questions was "often", coded with response code of 3); the minimum score is 0 (the household responded "no" to all occurrence questions, frequency-of-occurrence questions were skipped by the interviewer, and subsequently coded as 0 by the data analyst.) The higher the score, the more food insecurity (access) the household experienced. The lower the score, the less food insecurity (access) a household experienced.[x]

HFIAS Score<br><br>(0-27)	Sum of the frequency-of-occurrence during the past four weeks for the 9 food insecurity-related conditions<br><br>Sum frequency-of-occurrence question response code (Q1a + Q2a + Q3a + Q4a + Q5a + Q6a + Q7a + Q8a + Q9a)

Next, the ***indicator***, average Household Food Insecurity Access Scale Score, is calculated using the household scores calculated above.

Average HFIAS Score	Calculate the average of the Household Food Insecurity Access Scale Scoresxi<br><br>Sum of HFIAS Scores in the sample<br>————————————————<br>Number of HFIAS Scores (i.e., households) in the sample

5.4 Household Food Insecurity Access Prevalence

The final indicator is a categorical indicator of Food Insecurity Status.[xii] The Household Food Insecurity Access Prevalence (HFIAP) Status indicator can be used to report household food insecurity (access) prevalence and make geographic targeting decisions. The change in HFIAP can also be tabulated. For instance, if 60 percent of households are severely food insecure (access) at baseline and only 30 percent are severely food insecure (access) at the end of the program, the prevalence of household food insecurity (access) would have decreased by 30 percentage points (or by 50 percent). Because the average HFIAS score is a continuous variable, it is more sensitive to capturing smaller increments of changes over time than the HFIAP indicator. Therefore, the HFIAP indicator should be reported in addition to, rather than instead of, the average HFIAS Score for program monitoring and evaluation.

The HFIAP indicator categorizes households into four levels of household food insecurity (access): food secure, and mild, moderately and severely food insecure. Households are categorized as increasingly food insecure as they respond affirmatively to more severe conditions and/or experience those conditions more frequently.

A food secure household experiences none of the food insecurity (access) conditions, or just experiences worry, but rarely. A mildly food insecure (access) household worries about not having enough food sometimes or often, and/or is unable to eat preferred foods, and/or eats a more monotonous diet than desired and/or some foods considered undesirable, but only rarely. But it does not cut back on quantity nor experience any of three most severe conditions (running out of food, going to bed hungry, or going a whole day and night without eating). A moderately food insecure household sacrifices quality more frequently, by eating a monotonous diet or undesirable foods sometimes or often, and/or has started to cut back on quantity by reducing the size of meals or number of meals, rarely or sometimes. But it does not experience any of the three most severe conditions. A severely food insecure household has graduated to cutting back on meal size or number of meals often, and/or experiences any of the three most severe conditions (running out of food, going to bed hungry, or going a whole day and night without eating), even as infrequently as rarely. In other words, any household that experiences one of these three conditions even once in the last four weeks (30 days) is considered severely food insecure.

Table 5 below illustrates this categorization. The categorization scheme is designed to ensure that a household's set of responses will place them in a single, unique category.

Table 5. Categories of food insecurity (access)

Question	Frequency		
	Rarely<br>1	Sometimes<br>2	Often<br>3
1a	food secure	mildly food insecure	mildly food insecure
2a	mildly food insecure	mildly food insecure	mildly food insecure
3a	mildly food insecure	moderately food insecure	moderately food insecure
4a	mildly food insecure	moderately food insecure	moderately food insecure
5a	moderately food insecure	moderately food insecure	severely food insecure
6a	moderately food insecure	moderately food insecure	severely food insecure
7a	severely food insecure	severely food insecure	severely food insecure
8a	severely food insecure	severely food insecure	severely food insecure
9a	severely food insecure	severely food insecure	severely food insecure

- food secure

- moderately food insecure

- mildly food insecure

- severely food insecure

First, a HFIA category ***variable*** is calculated for each household by assigning a code for the food insecurity (access) category in which it falls. The data analyst should have coded frequency-of-occurrence as 0 for all cases where the answer to the corresponding occurrence question was "no" (i.e., if Q1=0 then Q1a=0, if Q2=0 then Q2a =0, etc.) prior to assigning the food insecurity (access) category codes. The four food security categories should be created sequentially, in the same order as shown below, to ensure that households are classified according to their most severe response.

HFIA category	Calculate the Household Food Insecurity Access category for each household. 1 = Food Secure, 2=Mildly Food Insecure Access, 3=Moderately Food Insecure Access, 4=Severely Food Insecure Access<br><br>HFIA category = 1 if [(Q1a=0 or Q1a=1) and Q2=0 and Q3=0 and Q4=0 and Q5=0 and Q6=0 and Q7=0 and Q8=0 and Q9=0]<br><br>HFIA category = 2 if [(Q1a=2 or Q1a=3 or Q2a=1 or Q2a=2 or Q2a=3 or Q3a=1 or Q4a=1) and Q5=0 and Q6=0 and Q7=0 and Q8=0 and Q9=0]<br><br>HFIA category = 3 if [(Q3a=2 or Q3a=3 or Q4a=2 or Q4a=3 or Q5a=1 or Q5a=2 or Q6a=1 or Q6a=2) and Q7=0 and Q8=0 and Q9=0]<br><br>HFIA category = 4 if [Q5a=3 or Q6a=3 or Q7a=1 or Q7a=2 or Q7a=3 or Q8a=1 or Q8a=2 or Q8a=3 or Q9a=1 or Q9a=2 or Q9a=3]

Next, the **prevalence** of different levels of household food insecurity (access) is calculated.

HFIA Prevalence	Percentage of households that fall in each food insecurity (access) category. For example: "Percentage of severely food insecure (access) households."<br><br>Example:<br><br>$\frac{\text{Number of households with HFIA category =4}}{\text{Total number of households with a HFIA category}} \times 100$<br><br>For example: "Percentage of severely food insecure (access) households"<br><br>$\frac{\text{Number of households with HFIA category =4}}{\text{Total number of households with a HFIA category}} \times 100$

The HFIS indicators presented in the tabulation plan above are useful for reporting food insecurity (access) prevalence, for making population level targeting decisions, and for examining the impact of program activities on overall food insecurity (access) or some dimension of it. The indicators are not intended, however, to be used to determine the causes of a problem or to guide a response—e.g., assessments of nutrition knowledge in order to design a behavior change intervention. Though the information generated from the application of the HFIAS can be used for geographical or population-based targeting, it is important to use caution if targeting resources at an individual or household level (i.e., as a program eligibility criterion) since administering subjective questions to a household in order to determine whether that particular household will receive a benefit can easily create respondent bias.

References

Alaimo K., Olson, C.M., Frongillo, E.A. "Importance of cognitive testing for survey items: An example from food security questionnaires." *Journal of Nutrition Education* 31:269-275, 1999.

Coates, Jennifer, Edward Frongillo, Robert Houser, Beatrice Rogers, Patrick Webb, and Park Wilde. "The Experience of Household Food Insecurity Across Cultures: What Have Measures Been Missing?" *Journal of Nutrition,* 2005 (forthcoming).

Coates, J. "Experience and Expression of Food Insecurity Across Cultures:

Practical Implications for Valid Measurement." Washington, D.C.: Food and Nutrition Technical Assistance Project, FHI 360, 2004.

Coates, Jennifer, Patrick Webb, and Robert Houser. "Measuring Food Insecurity: Going Beyond Indicators of Income and Anthropometry." Washington, D.C: Food and Nutrition Technical Assistance Project, FHI 360, 2003.

FAO. "Rome Declaration on World Food Security." World Food Summit, Rome: Food and Agriculture Organisation, 1996.

Food and Nutrition Technical Assistance (FANTA) Project. "Measuring Household Food Insecurity Workshop, April 15-16, 2004 Workshop Report." Washington, D.C: Food and Nutrition Technical Assistance Project, FHI 360, 2004.

Melgar-Quinonez, Hugo. "Testing Food Security Scales for Low-cost Poverty

Assessment- Draft Report." Davis, California: Freedom from Hunger, 2004.

Frongillo, Edward A., Siméon Nanama, and Wendy S. Wolfe. "Technical guide to developing a direct, experience-based measurement tool for household food insecurity." Washington, D.C.: Food and Nutrition Technical Assistance, FHI 360, 2004.

Frongillo, Edward A. and Simeon Nanama. "Development and validation of an experience-based tool to directly measure household food insecurity within and across seasons in northern Burkina Faso." Ithaca: Division of Nutritional Sciences, Cornell University, 2003.

Hamilton, William L., John T. Cook, William W. Thompson, Lawrence F. Buron, Jr. Edward A. Frongillo, Christine M. Olson, and Cheryl A. Wehler. "Household food security in the United States in 1995: Summary report of the food security measurement project." Washington, D.C.: United States Department of Agriculture, 1997.

Perez-Escamilla, Rafael, Ana Maria Segall-Correa, Lucia Kurdian Maranha, Maria de Fatima Archanjo Sampaio, Leticia Marin-Leon, and Giseli Panigassi. "An adapted version of the U.S. Department of agriculture food insecurity module is a valid tool for assessing household food insecurity in Campinas, Brazil." *Journal of Nutrition* 134:8 1923-1928, 2004.

Radimer, K. L., C. M. Olson, and C. C. Campbell. "Development of indicators to assess hunger." *Journal of Nutrition* 120:11 1544-1548, 1990.

Radimer, K. L., C. M. Olson, J. C. Greene, C. C. Campbell, and J. P. Habicht. "Understanding hunger and developing indicators to assess it in women and children." Journal of Nutrition Education 24:1 S36-S44, 1992.

United States Agency for International Development (USAID). "Policy Determination 19, Definition of Food Security, April 13, 1992." Washington, DC, 1992.

Webb, Patrick, Jennifer Coates, and Robert Houser. "Allocative responses to scarcity: Self-reported assessments of hunger compared with conventional measures of poverty and malnutrition in Bangladesh." Boston: Tufts University Friedman School of Nutrition Science and Policy, 2002.

Wehler, C., R. Scott, and J. Anderson. "The community childhood hunger identification project: A model of domestic hunger -- demonstration project in Seattle, Washington." Journal of Nutrition Education 24: 29S-35S, 1992.

Wilde, Parke E. "Differential response patterns affect food security prevalence estimates for households with and without children." Journal of Nutrition 134: 910-915, 2004.

Appendix 1: Key Informant Interview Guide

The Key Informant Interview Guide describes the type of discussion that is required in order to develop words/phrases, examples, and definitions that are adapted to the local context so that questions are understandable to survey respondents. Each question below, along with the probes beneath it, should be reviewed with a group of key informants. For each question, the words or words that should be tested with the key informants are bolded in brackets. Based on information from the key informants, modifications may be made to the questionnaire. Modifications may either be "phrases" (where the context-specific words are added directly in the body of the question), "definitions" (to be added directly after the question the first time a term, like "household", is used), and "examples" (to be added in italics after the question). The instruction following each question and set of probes below specifies whether the modification should be done as a phrase, definition, or example.

Q1: Did you worry that your [household] would not have enough food?

Probes:

- We would like to add an interviewer definition to clarify the way that a "household" is described in this culture.
- For instance, in some cultures "household" might be defined as "people who live together and share food from a common pot"
- Can you tell us how people here would commonly describe a household?

Based on the responses to the probes, an interviewer-provided definition is then added to the questionnaire.

Example Adapted Question (Q1):

Did you worry that your household would not have enough food?

By "household" we mean those of you that sleep under the same roof and take meals together at least four days a week.

Q2: Were you or any household member not able to eat the [kinds of foods you preferred] because of a [lack of resources]?

Probes:

- This question asks about one aspect of sufficient diet quality, which is having control over the kinds of foods that one eats.
- By "kinds of foods you preferred" we mean foods that food secure people eat that food insecure people cannot afford to eat.
- We would like to add interviewer-provided examples of different kinds of foods that are considered "preferred foods" in this culture.
- What are some examples of foods that food secure people eat that food insecure people cannot afford to eat?
- This question asks whether the preferred foods were inaccessible due to a "lack of resources."
- By "lack of resources" we mean not having money or the ability to grow or trade for the food.
- How do people here usually talk about a "lack of resources"?

Based on the responses to the probes, an interviewer-provided definition for "lack of resources", and an interviewer example for "kinds of foods you preferred", should be added to the questionnaire.

Example Adapted Question (Q2)

How often were you or any of your household members not able to eat the kinds of foods you preferred because of a lack of resources?

Whenever we say "lack of resources", we mean not having the means to get food, either through growing it, purchasing it, or trading for it.

- **Interviewer-provided example 1:** "Preferred foods" might include big fish, sweets, cake, etc.
- **Interviewer-provided example 2:** "Preferred foods" might include fruits bought from the market, eggs, meat etc.
- **Interviewer -provided example 3:** "Preferred foods" might include whole rice rather than broken rice.

Q3: Did you or any household member have to eat [a limited variety of foods] due to a lack of resources?

Probes:

- When we say “a limited variety of foods”, we want to mean an undesired monotonous diet for an extended period of days.
- We would like to add interviewer-provided examples of what an undesirable monotonous diet might be.
- What types of foods are included in a diverse diet in this culture?

Based on the responses to the probes, context specific <u>examples</u> of “just a few kinds of foods” should be added to the questionnaire.

Example Adapted Question (Q3)

Did you or any household member eat a limited variety of foods due to a lack of resources?

- **Interviewer -provided example 1:** “A limited variety of foods” might be tortilla and salt.
- **Interviewer -provided example 2:** “A limited variety of foods” might be rice and beans only.

Q4: Did you or any household member have to eat some foods [that you really did not want to eat] because of a lack of resources to obtain other types of food?

Probes:

- We would like to know whether the household had to eat food that it considered to be undesirable or socially unacceptable.
- We would like to add an interviewer-provided examples of different kinds of foods that poor, food insecure people may eat that are considered undesirable in this culture.
- Are there examples of such foods that could apply here?

Based on the responses to the probes, context specific <u>examples</u> of a “foods that you really did not want to eat” should be added to the questionnaire.

Example Adapted Question (Q4)

Did you or other members of your household have to eat some foods that you really did not want to eat because you lacked resources to obtain other types of food?

- **Interviewer-provided example 1:** “A food you really did not want to eat” might include wheat porridge, wild taro root, etc.
- **Interviewer -provided example 2: “** “A food you really did not want to eat” might include broken rice, wild grasses, discarded food, etc.

Q5: Did you or any other household member have to eat a smaller [meal] than you felt you needed because there was not enough food?

Probes:

- This question asks about having to eat less in a meal than the respondent thinks they should.
- The term "meal" is understood differently in different cultures. By "meal" we mean the major eating occasions (not including snacks).
- We would like to make sure that the word "meal" is understood this same way.
- How are can we express this same concept of "meal" in this language and culture?

Based on the responses to the probes, a context specific word or phrase meaning "meal" should be added to the body of the question in the questionnaire.

Example Adapted Question (Q5)

Did you or any household member eat less in either the morning or evening meal than you felt you needed because there was not enough food?

Q6: Did you or any household member have to eat [fewer meals in a day] because there was not enough food?

Probes:

- This question asks about eating "fewer meals in a day" than the social norm.
- We would like to make sure that the phrase "fewer meals in a day" is understood relative to the local norm, which you can help us define.
- How many meals a day do food secure people in this population usually eat during this time of year?
- Was there any period of time during the last four weeks (30 days) when the number of meals per day varied from the norm?

Based on the responses to the probes, a context specific phrase with the number of meals that food secure people usually eat should be added to the body of the question in the questionnaire.

Example Adapted Question (Q6)

Did you or any household member have to eat fewer than three meals in a day because there was not enough food?

Q7: Was there ever no food to eat of any kind in your household because of lack of resources to get food?

Probes:

- We would like to add a phrase here that clarifies the meaning of "no food to eat"
- By "no food to eat" we mean that the food was not available in the household and could not be accessed by the household's usual means (e.g. through purchase, from the garden or field, from storage, etc.).
- What are the terms that best describe the concept of not having food on hand and not being able to access food through the usual channels?

Based on the responses to the probes, a context specific phrase meaning "no food to eat" should be added to the body of the question in the questionnaire.

Example Adapted Question (Q7)

- **Example 1:** Did your household ever have no food on hand and there was no way of getting more?
- **Example 2:** Were your household food stores ever completely empty and there was no way of getting more?

Q8: Did you or any household member go to sleep at night hungry because there was not enough food?

Probe:

- We think this question may not require any adaptation. Do you agree?

Q9: Did you or any household member go a whole day and night without eating anything because there was not enough food?

Probe:

- We think this question may not require any adaptation. Do you agree?

Endnotes

[i] Three distinct variables are essential to the attainment of food security: 1) Food Availability: sufficient quantities of appropriate, necessary types of food from domestic production, commercial imports or donors other than USAID are consistently available to the individuals or are within reasonable proximity to them or are within their reach; 2) Food Access: individuals have adequate incomes or other resources to purchase or barter to obtain levels of appropriate food needed to maintain consumption of an adequate diet/nutrition level; and 3) Food Utilization: food is properly used, proper food processing and storage techniques are employed, adequate knowledge of nutrition and child care techniques exist and is applied, and adequate health and sanitation services exist (USAID Policy Determination, Definition of Food Security, April 13, 1992).

[ii] Questions relating to coping strategies to augment the household resource base were tested, but not incorporated into the US Household Food Security Survey. These items did not fit the statistical model of food insecurity when tested alongside items representing another dimension of the problem (Hamilton et al., 1997).

[iii] In April 2004, FANTA held a two-day workshop bringing together USAID staff, researchers and Title II and Child Survival and Health Grant representatives to discuss the development of a scale to measure the severity of household food insecurity (access). This workshop was instrumental in the development of the original set of questions. The workshop report is found at www.fantaproject.org.

[iv] Domains are defined as the most core experiences of food insecurity that are common across countries and cultures (Coates, et al., 2005).

[v] This guide represents a set of “best practices” based on current research. However, researchers continue to investigate the best form and function of HFIAS. There is a need for further testing based on use of the same set of questions across multiple field sites. Field validation will provide data to test the unidimensionality and universality of the scale empirically.

[vi] The U.S. Household Food Security Survey Module (US HFSSM) and some household food insecurity (access) scales created for other countries included sets of questions addressing the conditions of adults and children separately. Because adults tend to “buffer” children from the effects of food insecurity, evidence of child deprivation often reflects a very severe manifestation of household food insecurity (access). However, because child-referenced questions are not applicable to the entire population, the U.S. HFSSM relies on a statistical method of equating the responses of households with and without children. Due to the uncertain validity of this statistical approach (see Wilde, 2004) and the inability to draw conclusions about individual child hunger from a household measure, US officials are working to develop a separate child food insecurity scale. The set of model questions presented in this guide avoids these issues by asking about all household members- with the understanding that the HFIAS’s ability to discriminate between degrees of household food insecurity (access) at the most severe levels may be slightly compromised.

[vii] Applications of food insecurity scales have generally used either 12-month, 6 month, or 30 day recall periods (Coates, 2004). The choice of recall period should be based on the following considerations1) the

degree to which household food insecurity is likely to fluctuate over time, 2) the intended application of the data, and 3) the ability of the respondent to accurately remember behaviors and attitudes. The 30-day recall period is recommended here based on the following considerations arising from experience in several contexts. This HFIAS is expected to be used both in contexts with rapidly changing situations, where the primary interest is in detecting acute/ transitory insecurity, as well as in relatively stable situations, where the problem is one of chronic food insecurity. The shorter recall period can be used for either type of situation and is more likely to elicit accurate and reliable responses.

[viii] These dimensions differ slightly from the ones that form the basis of the U.S. Household Food Security Survey Module. Based on a cross-country literature review (Coates, 2004), participants at the FANTA workshop agreed that this list was more comprehensive, and better-represented commonalities of the food insecurity (access) experience in different cultures.

[ix] Tufts University researchers are analyzing the responses of males and females in the same household to determine the implications of relying solely on one or the other gender as the respondent. Meanwhile, since interviewing several members in each household is usually not cost-effective, the person in charge of food preparation appears to be a reasonable alternative.

[x] If the average HFIAS score at a project baseline was '4', what does that average HFIAS score mean in and of itself? Is a household with a score of '4' food secure or not? It turns out that this is not an easy question to answer, since the HFIS is designed to provide a continuous, rather than a categorical, indicator of food insecurity (access) that captures relative shifts in the situation over time. Instructions for calculating a categorical indicator of food insecurity (access) are provided in Section 5.

[xi] The US HFSS uses a statistical model called the "Rasch Model" to create a food insecurity (access) scale in which intervals are equal (e.g. a score of 4 is twice as food insecure as a score of 2). The additive approach described here is much simpler, but as a result one cannot assume that the intervals between 0-27 on the HFIS are *necessarily* equivalent, (i.e. that an increase in the score from 25 to 27 means the same thing as an increase from 18 to 20). For instance, it is not recommended that an average increase from 12 to 24 be reported as a "doubling of food insecurity", but rather as a "doubling of the food insecurity score." The difference between using an additive scale versus an interval scale may not be large, and additive scales are quite commonly used in research and operational applications despite this technical limitation.

[xii] To date, there is no universally accepted approach to setting these cut-off points. One approach suggested in this section. It is based on a number of assumptions. FANTA will work with academicians and program managers to analyze HFIAS data collected by a range of users to test the universality of the suggested approach empirically.
